# Reduced Monocyte and Neutrophil Infiltration and Activation by P-Selectin/CD62P Inhibition Enhances Thrombus Resolution in Mice

**DOI:** 10.1161/ATVBAHA.123.320016

**Published:** 2024-02-22

**Authors:** Julia B. Kral-Pointner, Patrick Haider, Petra L. Szabo, Manuel Salzmann, Mira Brekalo, Karl H. Schneider, Waltraud C. Schrottmaier, Christoph Kaun, Sonja Bleichert, Attila Kiss, Romana Sickha, Christian Hengstenberg, Kurt Huber, Christine Brostjan, Helga Bergmeister, Alice Assinger, Bruno K. Podesser, Johann Wojta, Philipp Hohensinner

**Affiliations:** Ludwig Boltzmann Institute for Cardiovascular Research (J.B.K.-P., P.L.S., K.H.S., A.K., R.S., K.H., H.B., B.K.P., J.W., P. Hohensinner), Medical University of Vienna, Austria.; Division of Cardiology, Department of Internal Medicine II (J.B.K.-P., P. Haider, M.S., M.B., C.K., C.H., J.W.), Medical University of Vienna, Austria.; Centre for Biomedical Research and Translational Surgery (P.L.S., K.H.S., A.K., H.B., B.K.P., P. Hohensinner), Medical University of Vienna, Austria.; Institute for Vascular Biology and Thrombosis Research (W.C.S., A.A.), Medical University of Vienna, Austria.; Division of Vascular Surgery, Department of General Surgery (S.B., C.B.), Medical University of Vienna, Austria.; Department of Medicine, Cardiology and Intensive Care Medicine, Wilhelminenhospital, Vienna, Austria (K.H.).; Medical Faculty, Sigmund Freud University, Vienna, Austria (K.H.).

**Keywords:** fibrinolysis, monocytes, neutrophils, thromboplastin, venous thrombosis

## Abstract

**BACKGROUND:**

Venous thromboembolism is a major health problem. After thrombus formation, its resolution is essential to re-establish blood flow, which is crucially mediated by infiltrating neutrophils and monocytes in concert with activated platelets and endothelial cells. Thus, we aimed to modulate leukocyte function during thrombus resolution post-thrombus formation by blocking P-selectin/CD62P-mediated cell interactions.

**METHODS:**

Thrombosis was induced by inferior vena cava stenosis through ligation in mice. After 1 day, a P-selectin-blocking antibody or isotype control was administered and thrombus composition and resolution were analyzed.

**RESULTS:**

Localizing neutrophils and macrophages in thrombotic lesions of wild-type mice revealed that these cells enter the thrombus and vessel wall from the caudal end. Neutrophils were predominantly present 1 day and monocytes/macrophages 3 days after vessel ligation. Blocking P-selectin reduced circulating platelet-neutrophil and platelet-Ly6C^high^ monocyte aggregates near the thrombus, and diminished neutrophils and Ly6C^high^ macrophages in the cranial thrombus part compared with isotype-treated controls. Depletion of neutrophils 1 day after thrombus initiation did not phenocopy P-selectin inhibition but led to larger thrombi compared with untreated controls. In vitro, P-selectin enhanced human leukocyte function as P-selectin-coated beads increased reactive oxygen species production by neutrophils and tissue factor expression of classical monocytes. Accordingly, P-selectin inhibition reduced oxidative burst in the thrombus and tissue factor expression in the adjacent vessel wall. Moreover, blocking P-selectin reduced thrombus density determined by scanning electron microscopy and increased urokinase-type plasminogen activator levels in the thrombus, which accelerated caudal fibrin degradation from day 3 to day 14. This accelerated thrombus resolution as thrombus volume declined more rapidly after blocking P-selectin.

**CONCLUSIONS:**

Inhibition of P-selectin-dependent activation of monocytes and neutrophils accelerates venous thrombosis resolution due to reduced infiltration and activation of innate immune cells at the site of thrombus formation, which prevents early thrombus stabilization and facilitates fibrinolysis.

**Highlights**

- From the caudal site, neutrophils followed by monocytes infiltrate the thrombus and their composition varies between caudal and cranial thrombus parts during vena cava ligation-induced thrombosis in mice.
- P-selectin-coated beads increase tissue factor expression in classical human monocytes and reactive oxygen species production by human neutrophils in vitro.
- P-selectin inhibition post-thrombus formation reduces migration of neutrophils and Ly6C^high^ macrophages toward the cranial thrombus part and also reduces oxidative stress in the thrombus and tissue factor expression in the adjacent vessel.
- P-selectin inhibition post-thrombus formation increases thrombus porosity, uPA levels, and fibrin degradation supporting thrombolysis.
- Thus, early inhibition of P-selectin after thrombosis reduces thrombus stability accelerating thrombus resolution.

Upon vessel injury, hemostasis induces wound sealing to prevent excessive blood loss. Thereby, platelets form a clot, which is stabilized by fibrin, generated by the activated coagulation cascade. However, besides this vital task, uncontrolled activation of the hemostatic system can cause detrimental conditions of thrombus formation.^1,2^ Intraluminal fibrin-rich thrombi are clinically recognized as deep vein thrombosis (DVT). DVT can trigger pulmonary embolism, which is summarized as venous thromboembolism and is the third most common cause of vascular mortality worldwide^3^ affecting around 900 000 people per year in the United States^4^ and 370 000 people per year in Europe.^5^ Rapid diagnosis and thrombus resolution are required to re-establish blood flow after DVT and reduce the risk of embolic events and postthrombotic syndrome. Anticoagulation is the standard treatment for DVT reducing the risk for pulmonary embolism, DVT reoccurrence, and thrombus growth; however, anticoagulation is accompanied by elevated bleeding tendency, lack of acute thrombus lysis and still bears a high risk for postthrombotic syndrome.^6^

Common causes of thrombus formation are venous blood stasis, a hypercoagulability state of the blood, or harmed endothelium, which can lead to platelet activation and accumulation, thereby initiating clot formation. Subsequently, immune cells are recruited, erythrocytes are captured and a fibrin-rich thrombus develops.^7^ Leukocytes on the one hand support thrombus growth as, for example, inflammatory monocytes and alternatively activated macrophages can express TF (tissue factor, thromboplastin) and release TF-bearing microvesicles.^8^ Neutrophils enhance thrombosis by the release of serine proteases, which can directly activate various coagulation factors^9^ as well as by neutrophil extracellular traps (NETs), a mesh of extracellular DNA decorated with histones and antimicrobial proteins from neutrophil granules.^10^ On the other hand, infiltrated leukocytes also promote thrombus resolution via the uptake of cellular remnants as well as via regulating fibrinolysis. During fibrinolysis plasmin, generated from plasminogen by uPA (urokinase-type plasminogen activator) or tPA (tissue-type plasminogen activator), cleaves fibrin into fibrin degradation products.^11^ Fibrinolysis is the most important step to restore blood flow.^7^ Monocytes/macrophages and neutrophils express uPA and its receptor uPAR as well as receptors for plasminogen, for example, Plg-R_KT_, which facilitate plasminogen cleavage.^9,12^ Additionally, neutrophils and monocytes/macrophages influence collagen remodeling by secreting matrix metalloproteases.^13^ Moreover, they release cytokines affecting thrombus resolution, which is likely augmented by fibrin itself.^7^ Hence, inflammatory cells are crucially involved in thrombus resolution processes. However, whether their role in thrombus resolution is beneficial or detrimental is still unknown.

Innate leukocytes bind to selectins and cellular adhesion molecules to extravasate to the site of inflammation.^14^ P-selectin (CD62P) is rapidly translocated on the surface of damaged endothelial cells and activated platelets as it is stored in Weibel-Palade bodies and α-granules, respectively. Exposed P-selectin initiates binding between leukocytes and endothelium or platelets^15^ allowing rolling of the cells, which then leads to firm adhesion and transmigration via interactions with E-selectin, intercellular adhesion molecule-1 and vascular cell adhesion molecule-1.^14^ Moreover, direct contact between platelets and leukocytes influences leukocyte effector capacity.^16^ Thus, targeting the accessibility of P-selectin might regulate leukocyte accumulation and function during thrombus resolution. It was shown that blocking P-selectin before triggering thrombosis reduces thrombus formation and ameliorates vein wall inflammation and vein recanalization.^17,18^ Furthermore, soluble P-selectin is associated with a procoagulant state.^19^ However, molecular mechanisms underlying these beneficial effects are not defined and it is not clear yet how blocking P-selectin affects especially the resolution of already existing thrombi. This would be of interest in the clinical setting as a monoclonal antibody, Inclacumab, targeting P-selectin is currently studied in a phase III trial testing its efficacy to prevent reoccurring vaso-occlusive crisis in sickle cell disease patients (NCT04927247). Hence, using an inferior vena cava (IVC) ligation model in mice, we were interested in how P-selectin, targeted by a murine-specific monoclonal antibody, influences and modulates the activation and infiltration of innate leukocytes during thrombus resolution and thereby modulates resolution of DVT. Our approach therefore allows for the understanding of the contribution of P-selectin to venous thrombus remodeling after the initial thrombus formation is complete. It should be emphasized, that a possible therapeutic inhibition of P-selectin after thrombus formation would clearly expand options in the clinical setting.

## METHODS

Data are available on request from the authors. Please see the Major Resources Table and the Expanded Methods in the Supplemental Material for information on the used materials and more details.

### Mice

The Animal Care and Use Committee of the Medical University of Vienna and the Austrian Ministry of Sciences approved all performed mouse experiments (BMBWF-66.009/0402-V/3b/2018, BMBWF-66.009/0258-WF/V/3b/2017).

For all in vivo experiments, 8- to 12-week-old C57BL/6J wild-type sex-matched mice were used. Predominantly, female mice were used for IVC ligation as the surgery was optimized for females due to a lower size/fat ratio and to be consistent with the 3R’s (Replacement, Reduction, Refinement) we kept the variability marginal. For experiments with P-selectin inhibition and neutrophil depletion also male mice were included for confirmation and indicated as green points in the figures (Figures S2, S4, and S6). Littermates were randomly assigned to treatment or control groups. The treatment group received the monoclonal rat anti-mouse P-selectin antibody (CD62P, RB40.34, 2.4 µg/g, BD Biosciences), while the control group received rat IgG1, λ isotype control (2.4 µg/g, BD Biosciences) intravenously, both diluted in sterile PBS. We could confirm that this previously published concentration^20^ was sufficient to block aggregates of platelets with neutrophils and monocytes after ex vivo activation with convulxin, with no additional effect by increasing the dose to 4.0 µg/g (data not shown).

### Vena Cava Ligation

Anesthetized mice were placed on a heating pad and a midline laparotomy incision was performed. By retracting the abdominal organs and bluntly dissecting the retroperitoneum the IVC was exposed. Side branches of IVC were sealed, then a 5-0 prolene suture (Eticon) was placed next to the IVC as spacer and with a 7-0 silk suture (Eticon) the IVC was ligated together with the prolene suture. Afterward, the prolene spacer was removed, leading to a stenosis, and the abdomen was seamed together. Thrombus development in mice was monitored by ultrasonography (Vevo 2100, MS550 transducer) on postoperative days 3, 7, 10, and 14 under isoflurane (1%–1.5%, Abbott Laboratories Ltd) anesthesia as described previously.^21^ The volume of the thrombus was measured and analyzed by Vevo Lab (FUJIFILM VisualSonics, Inc.). At the end of the experiment thrombi, adjacent vessel wall and whole blood were collected. Thrombus weight was determined by a precision balance and the length was measured by a ruler. Murine blood counts were determined by VetABC Hematology Analyzer (Scil animal care company).

### Neutrophil Depletion

Neutrophil depletion was performed according to Boivin et al.^22^ Mice were either treated intravenously with 25 µg (up to 30 g bodyweight) rat anti-Ly6G (lymphocyte antigen 6 complex locus G6D; clone 1A8, BioXCell) or isotype control (rat IgG2a, anti-trinitrophenol, clone 2A3, BioXCell). After 2 hours, both groups received an anti-rat antibody (50 µg/mouse, clone: MAR 18.5, BioXCell). Antibodies were diluted in sterile saline. After 3 hours, blood was sampled to validate neutrophil counts.

### Human Monocytes

Human peripheral blood mononuclear cells were isolated from monocyte- and lymphocyte-enriched blood obtained via leukapheresis chambers of healthy donors according to recommendations of the ethical board of the Medical University of Vienna including informed consent (approval number 1575/2014). Enriched blood was overlayed on lymphocyte separation medium 1077 (PromoCell) in 50-mL SepMate tubes (Stemcell Technologies) and after centrifugation (1200*g*, 10 minutes) supernatant was taken and cells were washed with PBS. Cells were centrifuged at 500*g* for 5 minutes and resuspended in RPMI1640 (Thermo Fisher Scientific) supplemented with 10% fetal bovine serum (FBS, Millipore) and penicillin, streptomycin, fungizone, and glutamine (all Lonza). Peripheral blood mononuclear cells were stimulated with or without P-selectin-coated, uncoated, or L-selectin-coated beads (1:800 diluted in PBS, 38.8 µg/mL beads) and TNF (tumor necrosis factor)-α (10 ng/mL, R&D Systems) for 4 hours or indicated time points. If indicated, beads were preincubated with 10 µg/mL anti-P-selectin blocking antibody (R&D systems) for 20 minutes at room temperature. Subsequently, cells were stained for flow cytometry to distinguish the different monocyte populations and activation markers.

### Human Neutrophils

Human neutrophils were isolated via the EasySep Direct Human Neutrophil Isolation Kit (Stemcell Technologies) from EDTA-anticoagulated whole blood of healthy donors according to recommendations of the ethical board of the Medical University of Vienna including informed consent (approval number, EK237/2004). Neutrophils were preincubated with or without P-selectin-coated beads (1:800 in PBS, 38.8-µg/mL beads) for 20 minutes at 37 °C, and then TNF-α (10 ng/mL, R&D Systems) was added for 4 hours and subsequently stained for flow cytometry.

### Generation of P-Selectin-Beads

For the generation of P-selectin-coupled beads polybead-carboxylate-microspheres (2 µm, 2.7% solids (w/v) Polysciences), polylink protein coupling kit (Polysciences), and recombinant human P-selectin protein (carrier free, Catalog no. ADP3-050, R&D Systems) were used. Coupling was performed according to the manufacturer’s protocol. About 3.1 mg microparticles were centrifuged (1000*g*, 10 minutes) resuspended in polylink coupling buffer twice before 21-mg carbodiimide was added for activation. After 15 minutes, 50-µg P-selectin was added and incubated for 120 minutes. Then, beads were centrifuged (1000*g*, 10 minutes) and washed in storage buffer twice and stored at 4 °C in 100-µL storage buffer. As controls uncoupled beads (without protein incubation) and L-selectin-coupled beads were generated using recombinant human L-selectin (R&D Systems).

### Statistics

Statistics were performed using GraphPad Prism Software 8.0, and data were depicted as box plots representing median, first and third quartiles, and minimum and maximum or as scatter plots in which horizontal lines denote mean. D’Agostino-Pearson omnibus normality test or Kolmogorov-Smirnov test if n<8 was applied to examine normal distribution. Data were analyzed by 2-tailed paired or unpaired *t* test or 2-tailed Mann-Whitney *U* test. Data with multiple predicting factors were evaluated by 1- or 2-way ANOVA with Sidak correction for multiple comparisons.

## RESULTS

### Blocking P-Selectin Accelerates Thrombus Resolution and Prevents Heterotypic Aggregates of Platelets and Innate Leukocytes

**Figure 1.**
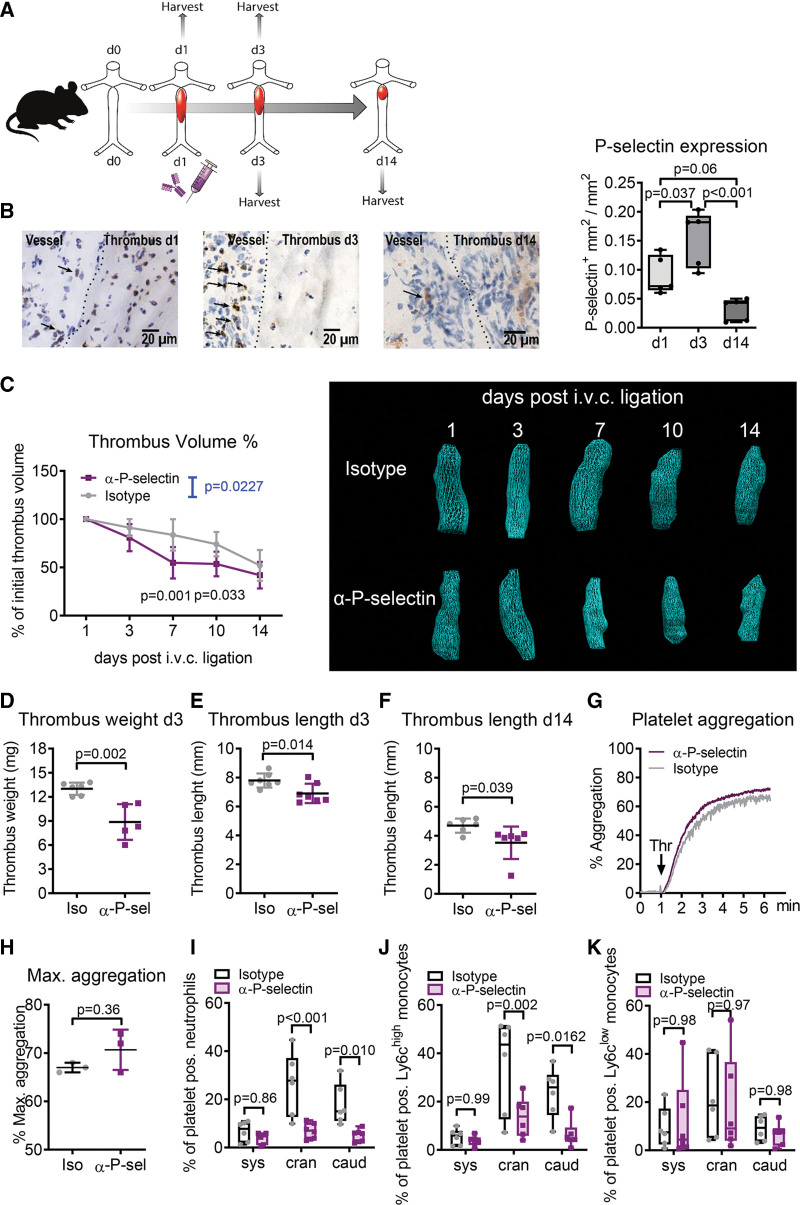
**Blocking P-selectin accelerates thrombus resolution. A**, Experimental design. The inferior vena cava (i.v.c.) of wild-type mice was ligated to induce venous stenosis followed by thrombus formation. The thrombus was analyzed either 1 or 3 days after surgery. Anti-P-selectin (α-P-sel) treatment was administered intravenously 1 day after vessel ligation and tissue was harvested 3 or 14 days after ligation. **B**, Sections of thrombus and surrounding vessel tissue were stained for P-selectin expression 1, 3, or 14 days after i.v.c. ligation. **C** through **F** and **I** through **J**, Wild-type mice were treated with a blocking P-selectin antibody or isotype (iso) 1 day after venous thrombus induction (n=5). **C**, The volume of the thrombus was measured by ultrasound over 14 days (n=6). *P* value of treatment-time-interaction was taken as an indicator for the effect of α-P-sel treatment and shown between label legends in blue. **D**, Thrombus weight of extracted thrombi at day 3, n=5 to 6. Length of extracted thrombi at (**E**) day 3 (n=7) and (**F**) 14 (n=6). **G** and **H**, Isolated murine platelets of mice treated with anti-P-selectin antibody or isotype were stimulated with thrombin (Thr, 230 mU/mL) and platelet aggregation was determined by light transmission aggregometry in vitro. A representative curve (**G**) and maximal aggregation (**H**) are given (n=3). **I** through **K**, Blood was sampled retro-orbitally (systemic, sys), cranially (cran), or caudally (caud) to the thrombus and analyzed by flow cytometry (n=6). Heterotypic aggregates between platelets and (**I**) neutrophils, (**J**) Ly6C^high^ monocytes, and (**K**) Ly6C^low^ monocytes were determined. Statistical analysis was performed by 1-way-ANOVA (**B**), 2-way ANOVA (**C**, **I**, **J**), unpaired (**D–F**), and paired (**H**) Student *t* test.

To assess the role of P-selectin in thrombus resolution, we measured P-selectin exposure during thrombus formation in mice. Therefore, the IVC was ligated together with a spacer, which was removed afterward, to induce venous stenosis in wild-type mice, and the formed thrombus was extracted after 1 or 3 days (Figure 1A). The sham procedure only demonstrated a weak P-selectin signal after 1 day when comparing to ligated mice on the same day (0.001%±0.002% P-selectin-positive area for sham-operated animals versus 0.090%±0.033% P-selectin-positive area in IVC-ligated mice; *P*=0.0079). Immunohistological staining revealed that P-Selectin was exposed in the vessel wall surrounding the thrombus and increased significantly from day 1 to day 3 and returned to lower levels again at day 14 (Figure 1B). This was paralleled by leukocyte influx into the thrombus (Figure S1). To study the effect of P-selectin-mediated leukocyte migration and activation on thrombus resolution, we treated mice with an antibody targeting murine P-selectin or isotype control antibody after 1 day, when the thrombus was already established (Figure 1A). Accordingly, we found that blocking P-selectin at day 1 accelerated thrombus resolution as the thrombus volume measured by ultrasound declined faster in mice treated with an anti-P-selectin antibody compared with isotype-treated littermates (Figure 1C). We further determined thrombus weight at day 3 as well as thrombus length at day 3 and 14, confirming a significant reduction in both parameters in anti-P-selectin-treated mice (Figure 1D through 1F). Since P-selectin is highly abundant on activated platelets, we checked if targeting P-selectin affects platelet aggregation in vitro. Thrombin-induced aggregation of platelets isolated from mice treated with anti-P-selectin antibody or isotype-treated control antibody was not different (Figure 1G and 1H), indicating that the antibody treatment did not interfere with platelet-platelet interaction during thrombus formation. Mice challenged with venous thrombosis had a significant drop in platelets over 3 days in both groups, while only little changes occurred in red and white blood cell counts or systemic MPO (myeloperoxidase) levels (Figure S2A through S2D). This drop in platelet count was unlikely due to systemic activation of hemostasis. Platelet markers for activation/aggregation (activated CD41/CD61; alpha IIb beta 3 integrin), degranulation (CD62P and CD63), as well as plasmatic thrombin-antithrombin complexes and CXCL4 (platelet factor 4) were unchanged after IVC ligation in both groups (Figure S2E through S2L). In contrast, platelets formed heterotypic aggregates with neutrophils and Ly6C^high^ (lymphocyte antigen 6 complex, locus C1) inflammatory monocytes at the caudal and cranial side of the thrombus (Figure 1I through 1J). Again, this was a local and not a systemic response as only low levels of heterotypic aggregates were detected in whole blood sampled from the retro-orbital plexus. Blocking P-selectin prevented aggregate formation between platelets and neutrophils or Ly6C^high^ inflammatory monocytes caudally and cranially to the thrombus, which suggests that P-selectin is essentially involved in modulating leukocyte function during thrombus resolution. Heterotypic aggregates of platelets with Ly6C^low^ patrolling monocytes were not blocked by antibody treatment, suggesting that other surface molecules mediate this interaction (Figure 1K). Of note, induction of venous thrombosis did neither significantly nor continuously elevate plasmatic inflammation markers (Figure S2M through S2Q).

### Neutrophils and Monocytes Enter the Thrombus Primarily Caudally

**Figure 2.**
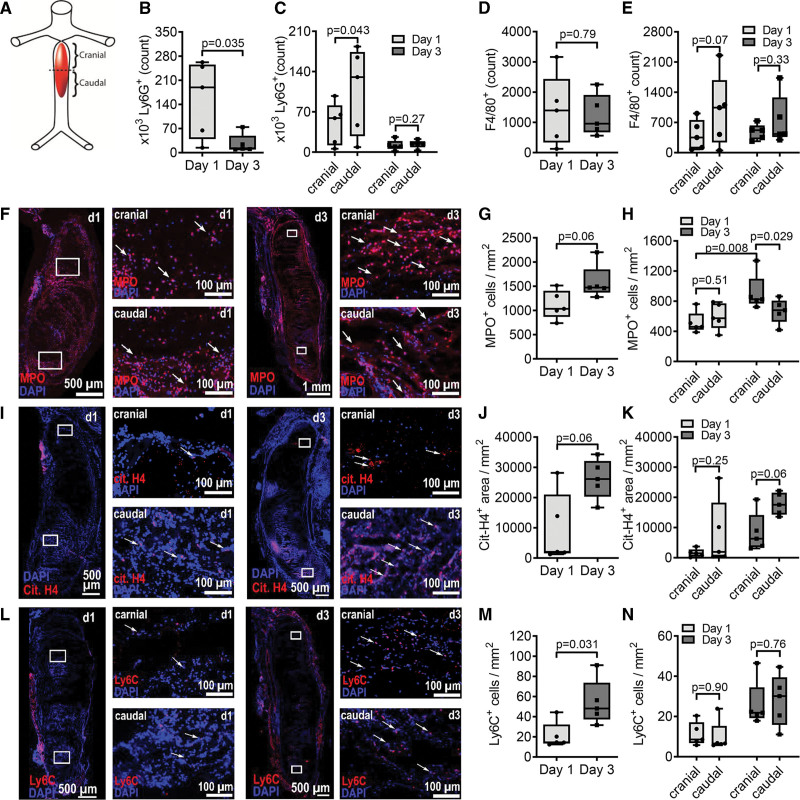
**Neutrophils enter the thrombus primarily caudally 1 day after thrombus formation and monocytes at day 3. A**, Illustration of thrombus partition. **B** through **N**, Thrombi were isolated 1 or 3 days after inferior vena cava ligation. **B** through **E**, Thrombi were bisected, homogenized, and analyzed by flow cytometry for the presence of CD45^+^ CD11b^+^ Ly6G^+^ neutrophils (**B** and **C**) or CD45^+^ CD11b^+^ F4/80^+^ macrophages (**D** and **E**). Total tissue (**B** and **D**) and bisected tissue (**C** and **E**) are depicted. **F** through **N**, Thrombi together with the adjacent vessel were extracted, cryopreserved, and generated sections were stained for MPO (myeloperoxidase; **F–H**); citrullinated histone H4 (cit. H4, **I–K**) or Ly6C (**L–N**). Representative images (20× magnification, **F**, **I**, **L**) and quantifications of positive cells within the thrombus are given in total thrombus (**G**, **J**, **M**) or bisected thrombus parts (**H**, **K**, **N**). Statistical analysis was performed by unpaired (**B**, **D**, **G**) and paired (**C** and **E**) Student *t* test, 2-way ANOVA (**H**, **K**, **N**) and Mann-Whitney *U* test (**J**, **M**). n=5.

To assess dynamic changes in the cellular composition during thrombus progression, we isolated thrombi and the adjacent vessel wall 1 or 3 days after vena cava ligation. The isolated tissue was bisected into a cranial and a caudal part to assess leukocyte localization (Figure 2A). Flow cytometric analysis revealed that significantly more neutrophils were found in the thrombus 1 day as compared with 3 days after thrombus induction and that significantly more neutrophils were present in the caudal part of the thrombus on day 1 (Figure 2B and 2C). A similar, albeit not significant trend was seen in the surrounding vessel wall (Figure S3A and S3B). Of note, in the vena cava of sham-operated mice used as a control, barely any neutrophils (117.3±45.23 cells) were detected. This indicates that neutrophils infiltrate the thrombus and possibly also the surrounding vessel from the caudal side and migrate toward the cranial part. We also found significantly more neutrophils in whole blood caudally of the thrombus on day 1 compared with day 3 (Figure S4A and S4B). Next, we measured MPO and citrullinated histone H4 in the thrombus as markers for neutrophil activation and NET formation, respectively (Figure 2F through 2K). We observed a trend toward increased levels of both MPO and citrullinated histone H4 on day 3 after thrombus induction as compared with day 1. This increase was significant when MPO levels in the cranial part of the thrombi were compared. These findings suggest that neutrophil recruitment predominantly occurred within the first 24 hours after ligation whereas neutrophil activation occurred at a later stage potentially involving also NET formation.

In contrast to neutrophils, significantly more macrophages were found 3 days after thrombus induction in the vessel wall as compared with day 1 (Figure S3C and S3D). In the vessel wall of sham-operated mice, only a few macrophages were detected (463.8±328.6 cells). In the thrombus, similar numbers of macrophages were detected on days 1 and 3 (Figure 2D). However, on day 1, more macrophages were found in the caudal site of the thrombus suggesting a caudal entry (Figure 2E). This is also indicated as both Ly6C^high^ inflammatory and Ly6C^low^ patrolling monocyte levels increased significantly in the blood caudal to the thrombus compared with the cranial site on day 1 (Figure S4A, S4C, and S4D). The macrophages present in the thrombus expressed Ly6C indicating their proinflammatory profile. The numbers of such proinflammatory macrophages were significantly higher in the thrombus on day 3 as compared with day 1. This significance, however, was lost, when cranial and caudal parts of the thrombi were compared (Figure 2L through 2N). Thus, our data indicate that the numbers of inflammatory macrophages increase time-dependently during thrombus formation from day 1 to day 3.

### Blocking P-Selectin Reduces Cranial Leukocyte Accumulation in the Thrombus

**Figure 3.**
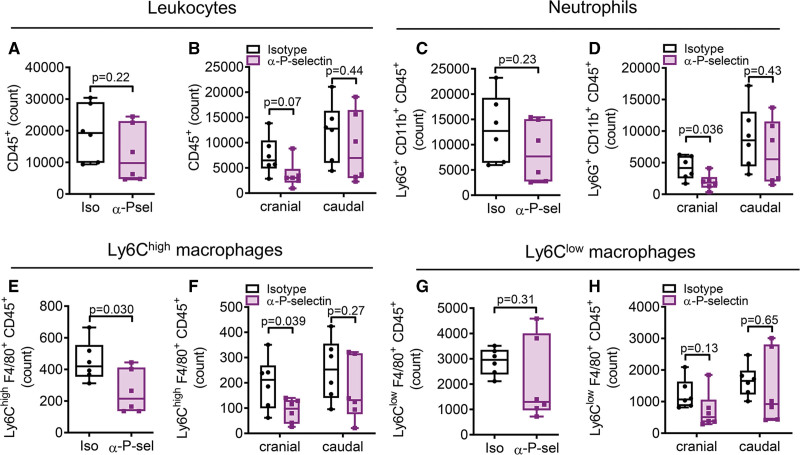
**Blocking P-selectin reduces leukocyte accumulation in the cranial part of the thrombus.** Thrombi of mice treated with anti-P-selectin antibody (α-P-sel) or isotype (iso) were isolated on day 3, bisected, and analyzed by flow cytometry. **A** and **B**, Leukocytes (CD45^+^), **C** and **D**, neutrophils (CD45^+^ CD11b^+^ Ly6G^+^), **E** and **F**, Ly6C^high^ macrophages (CD45^+^ F4/80^+^ Ly6C^high^), and **G** and **H**, Ly6C^low^ macrophages (CD45^+^ F4/80^+^ Ly6C^low^) were determined. Quantifications of these cells in total (**A**, **C**, **E**, **G**) or bisected (**B**, **D**, **F**, **H**) thrombus tissue are given. Statistical analysis was performed by unpaired Student *t* test. n=6.

Given the increase in thrombus resolution and reduction in platelet-leukocyte interaction when inhibiting P-selectin, we determined if P-selectin exposure is involved in the accumulation of innate leukocytes in the thrombus. We extracted thrombi and the surrounding vessel wall tissue 3 days after thrombus induction of mice with anti-P-selectin antibody or isotype treatment administered 1 day after thrombus induction. By bisecting the thrombus into 2 pieces, a cranial upper part and a caudal lower part, we localized cell influx. We found a trend for fewer leukocytes (*P*=0.07) and a significant decrease in neutrophils and Ly6C^high^ inflammatory macrophages only in the cranial part of the thrombus after P-selectin inhibition but not in the caudal part (Figure 3A through 3F). This finding indicates that blocking P-selectin at day 1 impeded migration and recruitment of the cells to the upper part 3 days after thrombus induction. However, numbers of Ly6C^low^ patrolling macrophages were neither changed in the cranial nor in the caudal thrombus part by the treatment as compared with control indicating that inhibition of P-selectin did not modulate infiltration of Ly6C^low^ patrolling monocytes into the thrombus (Figure 3G and 3H). This further underlines that blocking P-selectin did not influence platelet-Ly6C^low^ patrolling monocyte interactions.

Although P-selectin was detectable in the vessel surrounding the thrombus, we did not observe any changes in the leukocyte composition, including neutrophils, Ly6C^high^ inflammatory and Ly6C^low^ patrolling macrophages, in the vessel wall by the anti-P-selectin treatment (Figure S5A through S5H).

### Neutrophil Depletion After Thrombus Formation Decelerates Thrombus Resolution

**Figure 4.**
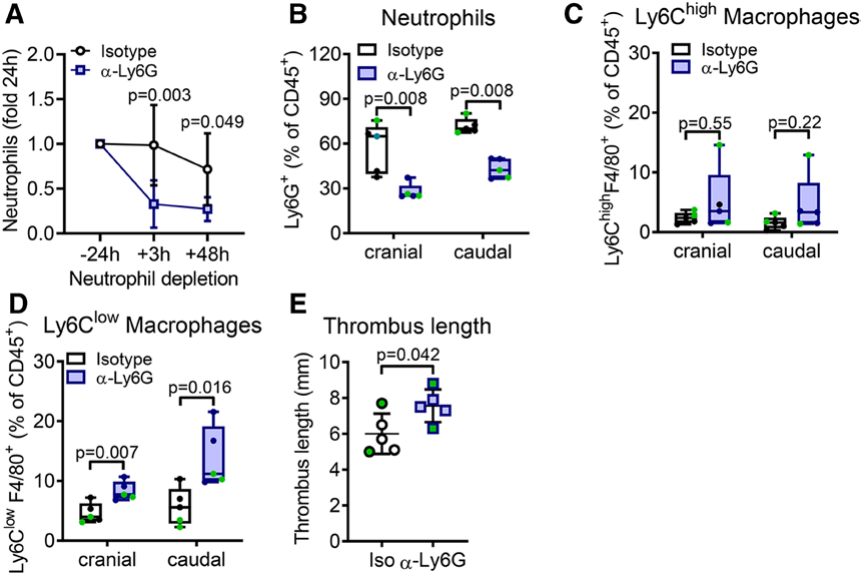
**Neutrophil depletion 1 day after thrombus formation increases Ly6C^low^ macrophages and thrombus length.** Mice were challenged with inferior vena cava ligation and after 1 day mice were treated with either an antibody against Ly6G to deplete neutrophils (alpha-Ly6G) or isotype control antibody (isotype, iso). **A**, Neutrophil (CD11b^+^ Ly6C^−^ cells) counts in whole blood were determined prior vena cava ligation, 3 and 48 hours post-neutrophil depletion via flow cytometry. **B** through **D**, Thrombi of neutrophil-depleted or control mice were isolated on day 3, bisected and analyzed by flow cytometry. **B**, Neutrophils (CD45^+^ CD11b^+^ Ly6G^+^), (**C**) Ly6C^high^ macrophages (CD45^+^ CD11b^+^ F4/80^+^ Ly6C^high^), and (**D**) Ly6C^low^ macrophages (CD45^+^ CD11b^+^ F4/80^+^ Ly6C^low^) were determined and depicted as percentage of total leukocytes. **E**, Thrombus length at day 3. Statistical analysis was performed by 2-way ANOVA (**A**), unpaired Student *t* test (**D** and **E**), and Mann-Whitney *U* test (**B** and **C**). Green data points indicate male mice. n=5.

As P-selectin blockage changed the cellular composition of the thrombus, we tested if enhanced thrombus resolution is mediated through the lack of neutrophils. Thus, we depleted neutrophils via an antibody against Ly6G 1 day after thrombus resolution similar to the application of P-selectin inhibition. Treatment efficiency was confirmed as circulating neutrophil counts were almost completely abolished after 3 hours and remained significantly reduced until harvest (Figure 4A), while monocytes and lymphocytes were not affected (Figure S6A through S6D). Inhibition overall led to a reduction in neutrophils within the thrombus (Figure 4B) with a similar distribution of Ly6C^high^ macrophages (Figure 4C) but a significant increase in Ly6C^low^ macrophages (Figure 4D). Overall, thrombus length increased when depleting neutrophils (Figure 4E) suggesting that depletion of neutrophils does not phenocopy P-selectin inhibition.

### P-Selectin Enhances Effector Functions of Human Neutrophils and Classical Monocytes

P-selectin not only mediates leukocyte extravasation but also regulates leukocyte effector function, which may also have pro-coagulatory and prothrombotic effects and thereby may hinder thrombus resolution. Direct binding of activated platelets to leukocytes affects inflammation, oxidative burst, and NET formation.^16^ Moreover, aggregate formation of platelets with monocytes increases monocytic TF expression,^23–25^ and interaction with neutrophils enhances superoxide production.^26^ Thus, we were interested in the direct effects of P-selectin on neutrophil and monocyte activation and if these effects are also targeted in vivo by blocking P-selectin. Therefore, we generated P-selectin-coated beads as P-selectin clustering enhances P-selectin function^23^ and verified P-selectin coupling by flow cytometry. We observed that around 47% of the beads were P-selectin positive compared with uncoated control beads (Figure S7A).

**Figure 5.**
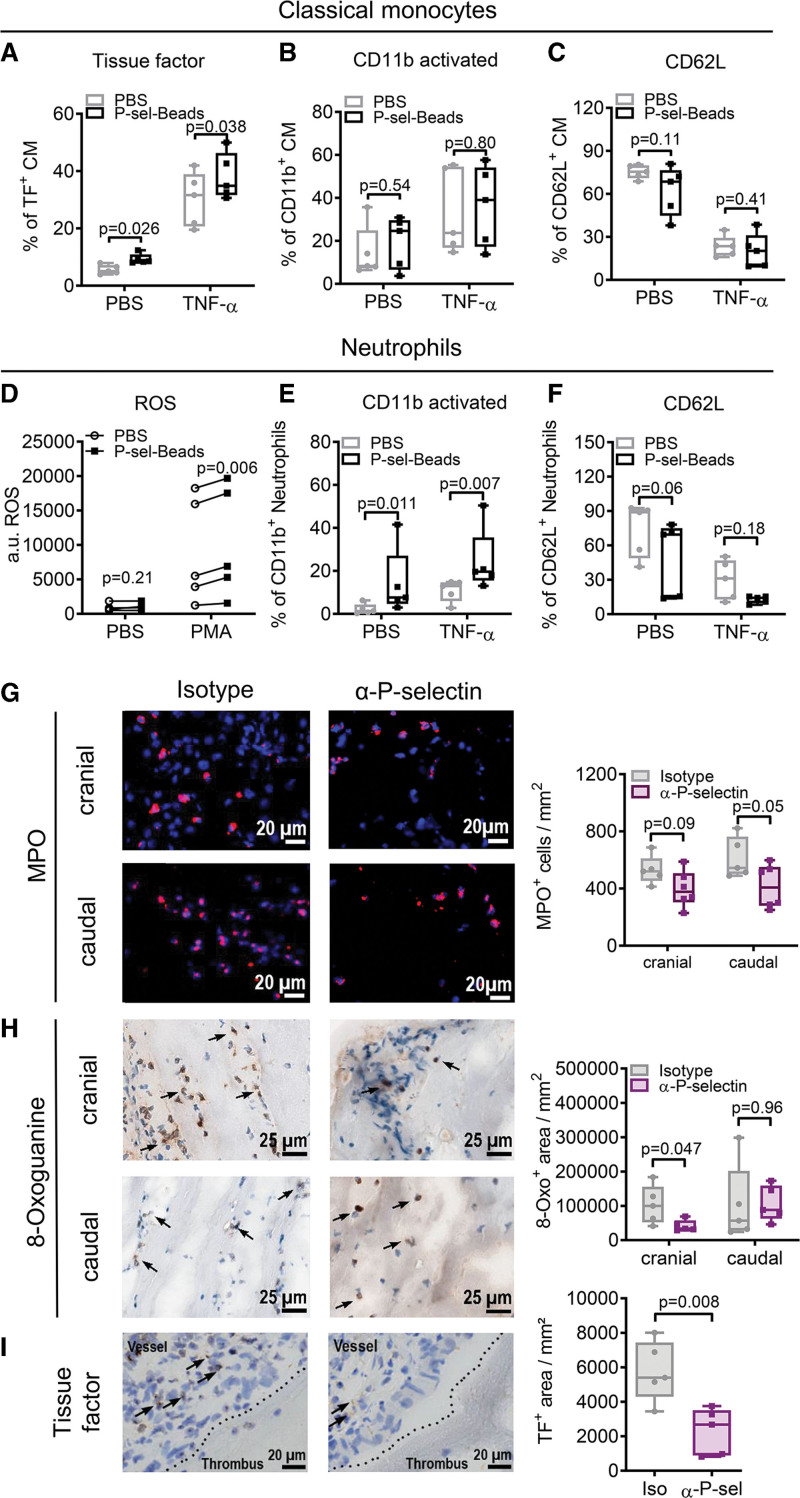
**P-selectin enhances effector functions of neutrophils and monocytes in vitro and in vivo. A** through **C**, Isolated human peripheral blood mononuclear cells were treated with P-selectin-beads or PBS and stimulated with TNF-α (tumor necrosis factor-α; 10 ng/mL) for 4 hours and analyzed by flow cytometry. Classical monocytes (CMs) were defined as CD11b^+^ CD14^+^ CD16^−^ and further analyzed for the expression of TF (tissue factor; **A**), CD11b activation (**B**), CD62L expression (**C**). **D** through **F**, Isolated human neutrophils were treated with P-selectin-beads (P-sel.-Beads) or PBS and stimulated with phorbol myristate acetate (PMA, 100 nmol/L) for 30 minutes (**D**) or with TNF-α (10 ng/mL) for 4 hours (**E** and **F**) and then reactive oxygen species (ROS; **D**), CD11b activation (**E**), and CD62L expression (**F**) were measured. **G** through **I**, Thrombi and surrounding vessel wall of mice treated with anti-P-selectin antibody (α-P-sel) or isotype 1 day after thrombus induction were isolated, cryopreserved, and sections were stained for MPO (myeloperoxidase, red; **G**), 8-oxoguanine (8-Oxo, brown; **H**) or TF (brown; **I**). Representative images and quantification of the thrombus (**G** and **H**) or vessel wall tissue (**I**) are given. Statistical analysis was performed by paired (**A–E**) and unpaired (**G–I**) Student *t* test and Wilcoxon (**F**). n=5. a.u. indicates arbitrary units.

Stimulating peripheral blood mononuclear cells with P-selectin-coated beads led to enhanced TF expression by classical monocytes (CMs) under basal conditions and after TNF-α stimulation (Figure 5A). A concentration of 38.8 µg/mL beads but not 19.4 µg/mL led to a significant induction of TF expression in CMs (Figure S7B). Pretreating the beads with a P-selectin blocking antibody (30 minutes, 10 µg/mL) significantly reduced TF enhancement by 19.6% compared with uncoated beads (*P*<0.05). To further verify a specific response, we also coated beads with L-selectin, which did not induce TF expression in CMs (Figure S7C). However, the conformational change of integrin CD11b toward its activated state CD11b and CD62L (L-selectin) shedding were left unaffected (Figure 5B and 5C) in this setting. Furthermore, P-selectin did not modulate TF expression, CD11b activation, nor CD62L shedding of intermediate monocytes (Figure S8A through S8C) and non-CMs (Figure S8D through S8F). Thus, we suggest that this interaction occurs primarily in an inflammatory setting as also neutrophil function was modulated. Binding of neutrophils to P-selectin-coated beads augmented reactive oxygen species (ROS) production upon Polymethyl acrylate stimulation (Figure 5D). ROS in turn can lead to further immune cell activation and increase blood coagulability.^27^ Also, CD11b activation was enhanced when neutrophils were costimulated with P-selectin-coated beads basally and after TNF-α stimulation (Figure 5E), and there was a trend of reduced CD62L expression suggesting an increase in shedding of CD62L under these conditions (Figure 5F). Thus, these data indicate an enhanced inflammatory response by neutrophils and CMs in the presence of P-selectin.

### Blocking P-Selectin Reduces Immune Cell Function

Given that P-selectin signaling had activating effects on neutrophils and monocytes, we next investigated possible attenuating effects on leukocytes by blocking P-selectin during thrombus resolution. Thus, we determined leukocyte activation markers in the thrombus of mice treated with an anti-P-selectin antibody or isotype control 1 day after thrombus induction. Analysis of histological sections revealed a trend for diminished MPO levels in the cranial (*P*=0.09) and caudal (*P*=0.05) part of thrombi treated with P-selectin blocking antibody (Figure 5G). Moreover, we observed a significant reduction in 8-oxoguanine, an indirect marker for ROS burden (Figure 5H), indicating a downregulation of oxidative stress mediated by neutrophil activation through P-selectin inhibition. Since neutrophils are prone to form NETs in thrombi, we also determined citrullinated histone H4. However, we did not find a significant decrease in citrullinated histone H4 levels by P-selectin blockage (Figure S9A), suggesting that NET formation was already initiated before P-selectin inhibition was applied.

As we measured elevated expression of TF upon P-selectin stimulation in monocytes, we also determined levels of TF in the vessel wall adjacent to the thrombus. We observed significantly less TF in the vessel wall after P-selectin inhibition as compared with isotype-treated control mice (Figure 5I). This points toward reduced monocyte/macrophage activation, which may in turn reduce the procoagulant state in these mice.

### Blocking P-Selectin Enhances Fibrinolysis

**Figure 6.**
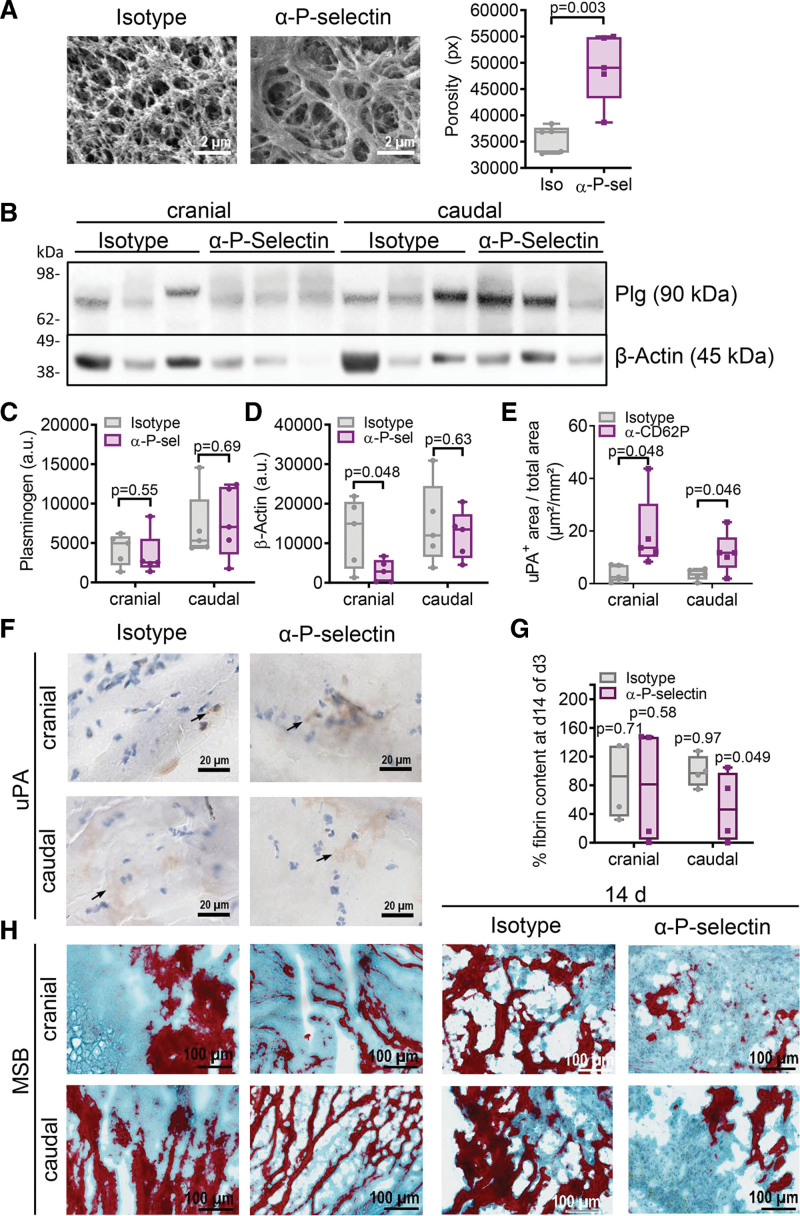
**Blocking P-selectin 1 day after thrombus formation enhances thrombus lysis.** Thrombi of mice treated with anti-P-selectin antibody (α-P-sel) or isotype at day 1 were isolated. **A**, Scanning electron microscopy (SEM) was performed of thrombus slices at day 3 and images were analyzed for thrombus porosity. **B** through **D**, Bisected thrombi were homogenized and analyzed for plasminogen (Plg; **C**) and β-actin (**D**) by Western blotting. A representative image and quantifications are given. **E** through **H**, Cryosections of thrombi were stained for uPA (urokinase-type plasminogen activator; brown) at day 3 (**E** and **F**) and for fibrin (red) and extracellular matrix (blue) via the Martius, Scarlet, and Blue stain (MSB) at day 3 (**left**) and day 14 (**right**; **G** and **H**). Representative images and quantifications are given. Percentage of fibrin content between day 3 (d3) and day 14 (d14) were compared and *P* values depicted. Statistical analysis was performed by unpaired Student *t* test. n=5 (day 3), n=4 (day 14).

As thrombus porosity can facilitate thrombus resolution by fibrinolytic factors,^28,29^ we employed scanning electron microscopy to investigate whether reduced leukocyte counts and activation seen under anti-P-selectin treatment would influence thrombus structure. Indeed, anti-P-selectin treatment significantly increased thrombus porosity (Figure 6A), which was equally increased in caudal and cranial sections (Figure S9B). To determine the availability of plasminogen/plasmin within the thrombus of treated and untreated animals, we determined plasminogen levels within the cranial and caudal part of day 3 thrombi. Our data indicate similar availability of plasminogen (Figure 6B and 6C) and further indirectly confirms our observation of reduced cellular density after P-selectin inhibition by demonstrating reduced β-actin levels, which were used to normalize for cellular input (Figure 6D). Since plasminogen needs to be cleaved to act fibrinolytically, we determined uPA distribution levels and observed significantly increased areas of uPA in the cranial and caudal thrombus part of anti-P-selectin-treated thrombi at day 3 (Figure 6E and 6F). Determining the fibrin content at day 3 and day 14 via the Martius, Scarlet, and Blue stain revealed a significant fibrin reduction caudally only in the anti-P-selectin-treated group, while no significant difference occurred in the isotype-treated group (Figure 6G and 6H). This demonstrates that uPA and increased porosity are required for efficient fibrinolysis and that blocking P-selectin favors these conditions.

## DISCUSSION

In this study, we used the IVC stenosis model that leads to the establishment of a laminar thrombus and is applied to study acute and chronic DVT. In this model, IVC ligation causes a reduction in blood flow that initiates thrombus development and mimics thrombi, which have been reopened on sites of vascular narrowing but does not mimic complete occlusion.^30^ The advantage of the applied stenosis model is that thrombosis is induced without chemical or physical damage to the vessel wall and thereby more closely mimics the situation in humans during DVT.^31^

Thrombus resolution is essential to re-establish blood flow, prevent embolic events and postthrombotic syndrome. Here, we show that blocking P-selectin on activated platelets and endothelial cells attenuates neutrophil and monocyte activation and reduces neutrophil and Ly6C^high^ monocyte accumulation in the cranial thrombus part, leading to increased thrombus porosity and uPA levels accelerating fibrin degradation. We thus provide novel mechanistic insight how P-selectin inhibition could ameliorate thrombus resolution (Graphical abstract).

Neutrophils and monocytes contribute decisively to thrombus development and resolution. Crawling and adhesion of neutrophils and monocytes to the venous endothelium initiates thrombus formation in the murine venous stenosis model.^32^ We could show for the first time that neutrophils and monocytes accumulate in the circulation caudally to the thrombus before they become equally distributed. Neutrophils and monocytes enhance intraluminal fibrin deposition by binding and activating coagulation factor XII as well as TF.^32,33^ Lack of peripheral neutrophils during IVC ligation reduces intrathrombus neutrophils and neutrophil activity, which substantially downregulates thrombus progression.^34^ Depletion of neutrophils reduces thrombus size in tumor-bearing mice.^35^ In contrast, lack of neutrophils during the repair process delayed venous thrombus resolution during venous stasis.^36^ Depletion of neutrophils within our model interestingly increased Ly6C^low^ macrophage levels and prolongated thrombus decay with reduced neutrophil content and similar Ly6C^high^ macrophages within the thrombus area as compared with controls. Given that Ly6C^low^ macrophages showed no alteration with a P-selectin blockade in our experimental setups, we suggest that the alteration observed within this cell type is independent of P-selectin modulation and a direct consequence of the missing neutrophils. We therefore speculate that this phenotype would also occur when combining neutrophil depletion and P-selectin inhibition shifting the balance within the thrombus to Ly6C^low^ macrophages. We suggest that this increase in Ly6C^low^ macrophages would not predominantly change the initial thrombus formation but might lead to altered resolution of the thrombus given the important role of Ly6C^low^ macrophages in thrombus resolution.^37^ Our data further confirm previous finding of the importance of Ly6C^high^ macrophages in the control of thrombus resolution given that the shift towards Ly6C^low^ macrophages was detrimental to thrombus resolution.^38^

Anti-P-selectin treatment 1 day after thrombus formation reduced neutrophils and Ly6C^high^ monocytes in the cranial thrombus part and was linked to increased TF availability within the thrombus periphery and therefore is directly linked to thrombus stability. Interestingly, a reduction of Ly6C^high^ monocyte-derived macrophages due to P-selectin blockage enhanced thrombus resolution suggesting that the stabilization of the thrombus by monocytes and macrophages is more detrimental to resolution than a reduced number of macrophages during the resolution process itself.

We could further demonstrate that stenosis-induced thrombosis causes binding of platelets to neutrophils and Ly6C^high^ monocyte in close proximity to the thrombus, which supports leukocyte extravasation and indicates platelet activation. An important interaction to mediate leukocyte infiltration is the binding of leukocyte PSGL-1 (P-selectin glycoprotein ligand) to endothelial or platelet P-selectin.^16,39,40^ Platelets capture neutrophils and subsequently inflammatory monocytes through CD40 binding to CD40L (CD154) and P-selectin binding to PSGL-1. The latter interaction triggers an ERK 1/2 (extracellular signal-regulated kinase) MAPK (mitogen-activated protein kinase)-dependent conformational change of CD11b/CD18 (β2 integrin Mac-1) into its high-affinity state to bind CD54 (ICAM-1 [intercellular adhesion molecule 1]), thereby enhancing transmigration.^40^ Our data show that leukocyte infiltration into the established thrombus is to some extent mediated by P-selectin. Preventive blocking of P-selectin by a specific antibody,^18,41^ recombinant PSGL-Ig,^17^ or an aptamer^42^ decreases vein wall inflammation,^18,41^ venous thrombus formation,^17,18^ and improves vein recanalization.^42^ Moreover, temporary IVC ligation for 48 hours and subsequent single treatment with recombinant PSGL-Ig reduces vein wall collagen, thrombus size, and mass.^43^ However, daily treatment of IVC stenosis with a P-selectin inhibitor starting 48 hours after ligation decreases vein wall stiffness, intimal thickening, and vein wall MCP-1 (monocyte chemotactic protein-1) levels without affecting thrombus mass in rats.^44^ Continuous administration of P-selectin aptamer starting 48 hours after thrombus induction diminished vein wall collagen content and improved vein recanalization.^42^ Treatment 6 hours post-venous stasis also leads to thrombus reduction.^17^ These studies emphasize that blocking P-selectin has a protective function in thrombus formation and resolution. We could confirm that a single application of a P-selectin blocking antibody 1 day after thrombus induction significantly accelerates thrombus resolution. Our results suggest that the observed effects of P-selectin inhibition are due to reduced infiltration of monocytes and reduced activation of both monocytes and neutrophils within the thrombus. Our data indicate that P-selectin directly mediates activation of neutrophils and monocytes as demonstrated by ROS generation and TF production. Reducing ROS might in turn affect thrombus stability as oxidation of fibrinogen makes it more susceptible to conversion into fibrin.^45^ In addition, oxidative modifications of inhibitors of the coagulation cascade such as of protein C,^46^ thrombomodulin,^47^ and TF pathway inhibitor^48^ can lead to their inactivation. Interestingly, lack of antioxidative protein paraoxonase-2 in mice leads to enhanced oxidative stress, TF activity, and platelet activation.^49^ We therefore propose that preventing activation of monocytes and neutrophils by inhibiting P-selectin-dependent processes is beneficial in venous thrombosis due to reduced infiltration and activation of these innate immune cells at the site of thrombus formation preventing early stabilization of the thrombus and facilitating thrombus resolution.

Thrombus resolution is accomplished by plasmin, which is generated from plasminogen via uPA or tPA. Upregulation of uPA levels via adenoviral gene transfer^50^ as well as monocyte-delivered adenoviral uPA^51^ demonstrated strong effects on thrombus resolution thereby demonstrating the importance of thrombus-localized uPA for thrombus resolution. Blocking P-selectin increased uPA levels and enhanced fibrin degradation further contributing to thrombus resolution. We therefore suggest that P-selectin inhibition supports thrombus resolution at least in part via increased uPA levels within the thrombus.

Activated platelets not only modulate neutrophil activation but contribute directly to thrombus formation. They further modulate coagulation by inducing TF expression in monocytes via P-selectin and PSGL-1.^23^ In line, P-selectin-expressing Chinese hamster ovary cells also cause TF expression in monocytes.^52^ Antibodies targeting TF or P-selectin decrease fibrin deposition on stents in vitro when perfused with whole blood.^53^ We could confirm that clustered P-selectin induced TF expression on monocytes under basal and inflammatory conditions. However, only CMs and not intermediate or non-CMs increased TF expression in the presence of P-selectin-coated beads. We suggest that our observation explains previous data, which showed that Ly6C^high^ monocytes are prothrombotic^38^ as only proinflammatory macrophages seem to be targets of P-selectin-mediated signaling.

It was previously shown that antiplatelet therapy reduces circulating TF procoagulant activity in patients with peripheral arterial disease.^54^ To target P-selectin directly 2 monoclonal antibodies crizanlizumab and inclacumab are currently available. Crizanlizumab has been conditionally authorized to treat painful vaso-occlusive crises in patients with sickle cell disease in the United States^55^ since 2019. In addition, it was demonstrated that crizanlizumab increases D-dimers, reduces prothrombin fragment 2.1, and induces endogenous thrombolysis in patients with COVID-19 (NCT04435184).^56^ Inclacumab is currently in phase 3 clinical trials to test its safety and efficacy also as a treatment for sickle cell disease (NCT04935879).^57^ Further, inclacumab ameliorates myocardial damage after percutaneous coronary intervention in patients with non-ST-segment elevation myocardial infarction (NCT01327183).^58,59^ However, inclacumab does not improve saphenous vein graft disease progression (NCT01245634).^60^ Our data suggest that inhibition of P-selectin has to start early after occlusion to prevent thrombus stabilization for optimal success. However, as we only used a single dose of P-selectin inhibition at 1 time point additional work is required concerning dosing and timing regimens for a possible therapeutic application.

In conclusion, we propose that a P-selectin-mediated cross talk between the endothelium, platelets, neutrophils, and monocytes in venous thrombosis results in infiltration of innate immune cells into the thrombus and their respective activation as indicated by their increased ROS production and TF expression. Elevated thrombus porosity and increased levels of uPA further contribute to accelerated thrombus resolution, suggesting beneficial effects of targeting P-selectin of already formed thrombi, which thus may provide new therapeutic options for the treatment of venous thrombosis.

## ARTICLE INFORMATION

### Acknowledgments

J.B. Kral-Pointner designed and performed experiments, evaluated data, compiled figures, wrote the article; P. Haider, P.L. Szabo, M. Salzmann, K.H. Schneider, W.C. Schrottmaier conducted and evaluated experiments; C. Kaun, M. Brekalo, S. Bleichert, and R. Sickha performed experiments; A. Kiss, C. Hengstenberg, C. Brostjan, H. Bergmeister, A. Assinger, B.K. Podesser, and K. Huber participated in experimental design and coordination; J. Wojta and P.H. supervised all research, designed experiments, interpreted results, and wrote the article. All authors read and approved the article. The authors thank Elena Pichler, Celine Nemethy, and Luis Pichelkastner for their excellent technical assistance and all animal caretakers for their invaluable assistance with animal experiments.

### Sources of Funding

This project was funded by the Austrian Science Fund (FWF; SFB-54 to J. Wojta and T-1251-B to J.B. Kral-Pointner) and by the Ludwig Boltzmann Institute for Cardiovascular Research.

### Disclosures

None.

### Supplemental Material

Expanded Materials and Methods

Figures S1–S9

Supplemental References

Major Resource Table

Uncropped Western blot images

## Supplementary Material


