# Supplementary material for: Reduced Monocyte and Neutrophil Infiltration and Activation by P-Selectin/CD62P Inhibition Enhances Thrombus Resolution in Mice
Source: Arterioscler Thromb Vasc Biol. 2024 Feb 22;44(4):954–68. doi: 10.1161/ATVBAHA.123.320016 (PMC11020038; doi:10.1161/ATVBAHA.123.320016)
Supplement: Supplementary file 1 [file atv-44-954-s001.pdf]

Supplemental Material

Valvular Prostaglandins are Elevated in Severe Human Aortic Valve Stenosis

Authors: Lucien G.J. Cayer MSc[1,2], Arun Surendran BE, MBA[3,4], Tobias Karakach PhD[1,5], Harold M. Aukema PhD[1,2,4], Amir Ravandi MD, PhD[2,4,6]

[1]Food and Human Nutritional Sciences, University of Manitoba, Winnipeg, MB, Canada;

[2]Canadian Centre for Agri-Food Research in Health and Medicine, Winnipeg, MB, Canada;

[3]Physiology and Pathophysiology, Rady Faculty of Health Sciences, University of Manitoba, Winnipeg, MB, Canada;

[4]Precision Cardiovascular Medicine Group, St. Boniface Hospital Research Centre, Winnipeg, MB, Canada;

[5]Pharmacology, Dalhousie University, Halifax, NS, Canada;

[6]Section of Cardiology, Max Rady College of Medicine, University of Manitoba, Winnipeg, MB, Canada.

Table S1.

Characteristics of healthy control patients included in the plasma oxylipins analysis.

	Control<br>n=44
Age	59.7 (10.9)
Sex (Male)	24 (54.5%)
Height (cm)	167 (9.84)
Weight (kg)	86.3 (22.9)
BSA (m^2)	1.99 (0.294)
BMI	30.9 (7.68)
Hypertension	19 (43.2%)
History of Smoking	9 (20.5%)
Current	5 (11.4%)
Previous	4 (9.09%)
Never	35 (79.5%)
Medication	
ACE inhibitors or ARBs	8 (18.2%)
ASA	6 (13.6%)
Betablocker	8 (18.2%)
Statin	7 (15.9%)
Coronary artery bypass graft	0 (0%)
Coronary artery disease	1 (2.27%)
Diabetes	
Type 1	0 (0%)
Type 2	8 (18.2%)
Dyslipidemia	10 (22.7%)
Chronic renal failure	1 (2.27%)
End-stage renal disease on hemodialysis	0 (0%)
Family history of cardiovascular disease	4 (9.09%)
History of congestive heart failure	3 (6.82%)
History of peripheral vascular disease	1 (2.27%)
ST-elevation myocardial infarction	0 (0%)
Non-ST-elevation myocardial infarction	0 (0%)
NYHA Classification	
Class I	28 (50.9%)
Class II	8 (14.6%)

Class III	1 (1.82%)
Class IV	0 (0%)
Obstructive valvular heart disease	5 (9.09%)
Percutaneous coronary intervention	2 (3.64%)
History of Smoking	14 (25.5%)
Current	7 (12.7%)
Previous	7 (12.7%)
Never	41 (74.5%)
Stroke	3 (5.45%)

Continuous variables are given as mean (standard deviation) and categorical variables are given as count (proportion), Abbreviations: ACE, angiotensin-converting enzyme; ARBs, angiotensin II receptor blockers; ASA, Acetylsalicylic Acid; BMI, body mass index; BSA, body surface area; NYHA, New York Heart Association.

Table S2.

The complete list of deuterated internal standards and oxylipins scanned for.

Analyte	Q1 Mass (Da)	Q3 Mass (Da)	Retention time (min)	LLOD (ng)	ULOD (ng)
Internal standards					
(d4) 6k-PGF1α	373	167	5.66	-	-
(d4) TXB2	373	173	6.97	-	-
(d4) PGF2α	357	197	7.55	-	-
(d4) PGE2	355	275	7.70	-	-
(d4) PGD2	355	275	8.08	-	-
(d4) 8-iso-PGF2αIII	357	197	6.81	-	-
(d4) 15d-PGJ2	319	203	14.72	-	-
(d4) dhk-PGF2α	357	295	8.95	-	-
(d4) LTB4	339	197	12.34	-	-
(d8) 5-HETE	327	116	17.33	-	-
(d8) 12-HETE	327	184	16.64	-	-
(d8) 15-HETE	327	226	16.11	-	-
(d6) 20-HETE	325	281	15.14	-	-
(d11) 8,9-DiHETrE	348	127	15.02	-	-
(d11) 11,12-DiHETrE	348	167	14.41	-	-
(d11) 14,15-DiHETrE	348	207	13.67	-	-
(d4) 9-HODE	299	172	16.03	-	-
(d4) 13-HODE	299	198	15.87	-	-
(d7) 5-oxoETE	323	279	17.74	-	-
(d5) RvD1	380	141	8.81	-	-
(d4) 9,10-diHOME	317	203	13.42	-	-
(d4) 12,13-diHOME	317	185	12.84	-	-
Oxylipins					

PGD2	351	271	8.12	0.00138	8.89
15d-PGD2	333	271	12.24	0.000395	8.89
dhk-PGD2	351	207	9.50	0.000395	8.89
15k-PGD2	349	235	8.95	-	-
PGJ2	333	189	10.17	0.000395	8.89
15d-PGJ2	315	203	14.74	0.00484	8.89
11β-PGF2α	353	335	7.01	0.00484	8.89
11β-dhk-PGF2α	353	113	8.47	0.00484	8.89
2,3-dinor-11β-PGF2α	325	227	5.94	0.00484	8.89
PGK2	349	249	8.84	0.00138	8.89
PGE2	351	271	7.71	0.00138	8.89
PGA2	333	271	10.00	0.000395	8.89
PGB2	333	271	10.25	0.00138	8.89
bicyclo-PGE2	333	175	10.86	0.00484	8.89
dhk-PGE2	351	207	8.72	0.0169	8.89
11β-PGE2	351	271	7.75	0.0169	8.89
15k-PGE2	349	235	8.09	0.00138	8.89
6k-PGF1α	369	245	5.68	0.00138	8.89
2,3-dinor-6k-PGF1α	341.2	261	6.90	-	-
6k-PGE1	367	331	5.95	0.000395	2.54
6,15-dk-dh-PGF1α	369	267	6.90	0.0169	8.89
PGF2α	353	193	7.56	0.000395	8.89
dh-PGF2α	355	283	8.25	-	-
dhk-PGF2α	353	291	8.97	0.000113	8.89
15k-PGF2α	351	219	8.02	0.00138	8.89
TXB2	369	169	6.94	0.00138	2.54
2,3-dinor-TXB2	341	137	5.92	0.00484	8.89
11d-TXB2	367	305	7.84	0.000395	8.89
12-HHTrE	279	217	13.82	0.00138	8.89
PGD1	353	235	8.03	0.000395	2.54
PGK1	351	251	9.09	0.00138	8.89

PGE1	353	235	7.90	0.000395	8.89
15k-PGE1	351.2	237	8.40	0.000395	2.54
PGF1α	355	293	7.54	0.000395	8.89
15k-PGF1α	353	221	8.19	0.000395	8.89
TXB1	371	171	6.60	0.00138	2.54
dihomo-PGD2	379	299	10.03	-	-
dihomo-15d-PGD2	361	299	14.31	-	-
dihomo-PGJ2	361	299	12.76	-	-
dihomo-PGE2	379	299	9.70	-	-
dihomo-PGF2α	381	337	9.50	0.00138	8.89
PGD3	349	269	7.09	0.00484	8.89
PGE3	349	269	6.75	0.00484	8.89
Δ17-6k-PGF1α	367	163	4.98	0.000113	8.89
PGF3α	351	193	6.54	0.00138	8.89
TXB3	367	169	6.03	0.00138	2.54
9-HODE	295	171	16.11	0.000113	8.89
9-oxoODE	293	185	16.38	0.00484	8.89
13-HODE	295	195	15.94	0.000113	8.89
13-oxoODE	293	167	16.06	0.00484	8.89
9,10,13-triHOME	329	171	7.65	0.000113	8.89
9,12,13-triHOME	329	211	7.53	0.000113	8.89
13-HOTrE-γ	293	193	15.08	0.00138	8.89
5-HETrE	321	205	18.91	0.00138	8.89
8-HETrE	321	157	17.30	0.000395	8.89
15-HETrE	321	221	16.88	0.000395	8.89
5-HETE	319	115	17.46	0.000113	8.89
5-oxoETE	317	203	17.84	0.000113	8.89
5,15-diHETE	335	201	11.92	0.00138	8.89
8-HETE	319	155	16.76	0.000395	8.89
9-HETE	319	123	17.06	0.00138	8.89
11-HETE	319	167	16.54	0.000395	8.89

12-HETE	319	135	16.72	0.00138	8.89
12-oxoETE	317	153	16.76	0.0169	8.89
tetranor-12-HETE	265	109	13.72	0.000113	8.89
15-HETE	319	175	16.17	0.00138	8.89
15-oxoETE	317	113	16.30	0.000113	8.89
8,15-diHETE	335	235	11.34	0.00484	8.89
6t-LTB4	335	195	11.96	0.00138	8.89
6t, 12-epi-LTB4	335	195	12.08	0.000395	8.89
5,6-diHETE	335	115	15.72	0.000395	8.89
LTB4	335	195	12.40	0.000395	8.89
12-oxo-LTB4	333	179	13.08	0.000395	8.89
20oh-LTB4	351	195	5.92	0.00138	8.89
20cooh-LTB4	365	195	5.77	0.00484	8.89
12-epi-LTB4	335	195	12.42	0.000395	8.89
LTC4	624	272	12.50	0.00138	8.89
LTD4	495	177	9.60	0.00138	8.89
LTE4	438	333	11.47	0.000395	8.89
14,15-LTC4 (EXC4)	624	272	7.80	-	-
14,15-LTD4 (EXD4)	495	177	11.34	-	-
14,15-LTE4 (EXE4)	438	333	9.30	-	-
6(R)-LXA4	351	217	8.85	0.00138	8.89
6(S)-LXA4	351	115	9.11	0.000395	8.89
15(R)-LXA4	351	165	8.85	-	-
LXB4	351	221	7.87	0.00138	8.89
HXA3	335	195	14.80	-	-
HXB3	335	183	15.00	-	-
15-oxoEDE	321	223	17.99	0.00138	8.89
9-HOTrE	293	171	14.68	0.00138	8.89
9-oxoOTrE	291	185	15.17	0.000113	8.89
13-HOTrE	293.4	195	14.75	0.00138	8.89
13-oxoOTrE	291	247	15.00	0.207	8.89

LXA5	349	215	7.82	0.00138	8.89
RvE1	349	195	5.84	0.000395	8.89
5-HEPE	317	115	16.04	0.00138	8.89
8-HEPE	317	155	15.50	0.00138	8.89
9-HEPE	317	149	15.67	0.00138	8.89
11-HEPE	317	167	15.28	0.000395	8.89
12-HEPE	317	179	15.47	0.00138	8.89
15-HEPE	317	219	15.16	0.0592	8.89
RvD1	375	141	8.93	0.000113	8.89
RvD2	375	175	8.04	0.00484	8.89
RvD5	359.2	199	11.78	0.000395	8.89
PD1	359	153	11.15	-	-
15t-PD1	359	153	11.40	-	-
4-HDoHE	343	101	17.77	0.00138	8.89
7-HDoHE	343	141	16.92	0.000395	8.89
8-HDoHE	343	109	17.06	0.00138	8.89
10-HDoHE	343	153	16.53	0.000113	8.89
11-HDoHE	343	149	16.70	0.00138	8.89
13-HDoHE	343	221	16.34	0.00138	8.89
14-HDoHE	343	205	16.45	0.00138	8.89
16-HDoHE	343	233	16.14	0.000395	8.89
17-HDoHE	343	245	16.24	0.0169	8.89
10(S),17(S)-DiHDoHE (PDX)	359	153	11.57	0.00138	8.89
4k-DHA	341	297	18.10	0.00138	8.89
17k-DHA	341	297	16.43	0.0169	8.89
7(R)-Maresin-1	359	177	11.79	0.00138	8.89
17k-DPA	343	247	16.99	0.00484	8.89
9,10-EpOME	295	171	17.55	0.000395	8.89
9,10-diHOME	313	201	13.48	0.000395	8.89
12,13-EpOME	295	195	17.29	0.00138	8.89

12,13-diHOME	313	183	12.92	0.000113	8.89
5,6-EpETrE	319	191	18.33	0.000395	8.89
5,6-DiHETrE	337	145	15.91	0.00138	8.89
8,9-EpETrE	319	155	18.05	0.00138	8.89
8,9-DiHETrE	337	127	15.13	0.00138	8.89
11,12-EpETrE	319	167	17.78	0.00138	8.89
11,12-DiHETrE	337	167	14.50	0.00138	8.89
14,15-EpETrE	319	175	17.33	0.00484	8.89
14,15-DiHETrE	337	207	13.77	0.000113	8.89
9,10-EpODE	293	171	16.40	-	-
9,10-diHODE	311	201	11.88	-	-
12,13-EpODE	293	183	16.30	0.000395	8.89
12,13-diHODE	311	183	11.40	0.000113	8.89
15,16-EpODE	293	235	16.00	-	-
15,16-diHODE	311	223	11.53	-	-
14,15-EpETE	317	207	16.39	0.000113	8.89
14,15-diHETE	335	207	12.31	0.00484	8.89
17,18-EpETE	317	259	16.00	0.00138	8.89
17,18-diHETE	335	247	11.76	0.00484	8.89
16,17-EpDoPE	343	193	17.58	0.0169	8.89
16,17-DiHoDPE	361	233	14.14	0.000395	8.89
19,20-EpDoPE	343	241	17.09	0.00484	8.89
19,20-DiHDoPE	361	229	13.62	0.00484	8.89
16-HETE	319	189	15.62	0.000395	8.89
17-HETE	319	247	15.51	0.00138	8.89
18-HETE	319	261	15.40	0.00138	8.89
19-HETE	319	231	15.03	0.0592	8.89
20-HETE	319	245	15.17	0.00138	8.89
20cooh-AA	333	271	14.80	0.00484	8.89
18-HEPE	317	215	14.66	0.00138	8.89
20-HDoHE	343	241	15.88	0.00484	8.89

2,3-dinor 8-iso-PGF2α	325	237	5.55	0.00138	8.89
8-iso-15k-PGF2β	351	219	7.28	0.0169	8.89
5-iso-PGF2αVI	353	115	7.44	0.000395	8.89
8-iso-PGF2αIII	353	193	6.81	0.00484	8.89
8-iso-PGF3α	351	307	5.82	-	-
9-Nitrooleate	326	168	19.44	-	-
10-Nitrooleate	326	169	19.44	-	-
15d-PGA2	315	271	15.31	0.0592	8.89

LLOD and ULOD values were determined using 40 µL on the column of standards in MS grade water: acetonitrile: acetic acid (70:30:0.02; v/v/v). Abbreviations: AA, arachidonic acid; DHA, docosahexaenoic acid; DiHDoPE, dihydroxy-docosapentaenoic acid; DiHETE, dihydroxy-eicosatetraenoic acid; DiHETrE, dihydroxy-eicosatrienoic acid; DiHODE, dihydroxy-octadienoic acid; DiHOME, dihydroxy-octadecaenoic acid; DPA, docosapentaenoic acid; EpDoPE, epoxy-docosapentaenoic acid; EpETE, epoxy-eicosatetraenoic acid; EpETrE, epoxy-eicosatrienoic acid; EpODE, epoxy-octadecadienoic acid; EpOME, epoxy-octadecaenoic acid; HDoHE, hydroxy-docosahexaenoic acid; HEPE, hydroxy-eicosapentaenoic acid; HETE, hydroxy-eicosatetraenoic acid; HETrE, hydroxy-eicosatrienoic acid; HHTrE, hydroxyheptadecatrienoic acid; HODE, hydroxy-octadecadienoic acid; HOTrE, hydroxy-octadecatrienoic acid; HX, hepoxilin; LA, linoleic acid; LLOD, lower limit of detection; LT, leukotriene; LX, lipoxin; oxoEDE, oxo-eicosadienoic acid; oxoETE, oxo-eicosatetraenoic acid; oxoODE, oxo-octadecadienoic acid; oxoOTrE, oxo-octadecatrienoic acid; P, protectin; PG, prostaglandin; Rv, resolvin; triHOME, trihydroxy-octadecenoic acid; TX, thromboxane; ULOD, upper limit of detection.

Table S3.

Aortic valve leaflet oxylipins by aortic valve stenosis severity group determined by patient transvalvular mean pressure gradient.

		Aortic Valve Leaflet Oxylipins (µg/g protein)				
Oxylipin	Enzyme	Mild n=15	Moderate n=39	Severe n=45	*P* value	Adjusted *P* values
**LA oxylipins**						
9,10-diHOME	CYP	0.107±0.0461	0.156±0.0266	0.197±0.0292	0.060	>0.99
12,13-diHOME	CYP	0.119±0.0580[b]	0.207±0.0363[ab]	0.232±0.0387[a]	0.041	>0.99
LA CYP total		0.226±0.104[b]	0.363±0.0615[ab]	0.429±0.0672[a]	0.023	0.14
9-HODE	LOX	70.2±42.9[b]	121±14.8[a]	174±29.8[a]	<0.001	0.002
9-oxoODE	LOX	26.7±7.84[b]	55.6±7.63[a]	66.3±10.8[a]	0.001	0.049
13-HODE	LOX	77.8±53.1[b]	122±15.2[a]	183±32.4[a]	<0.001	0.002
13-oxoODE	LOX	57.8±24.5[b]	125±17.6[a]	178±38.7[a]	<0.001	0.008
(9,10,13- & 9,12,13-) triHOME	LOX	1.22±0.656[b]	2.33±0.325[a]	2.69±0.434[a]	<0.001	0.023
LA LOX total		234±128[b]	426±52.4[a]	604±106[a]	<0.001	<0.001
LA total		234±128[b]	426±52.4[a]	604±106[a]	<0.001	<0.001
**GLA oxylipins**						
13-HOTrE-γ	LOX	0.304±0.152[b]	0.936±0.116[a]	1.12±0.168[a]	<0.001	<0.001
**DGLA oxylipins**						
8-HETrE	LOX	1.87±1.22[b]	3.25±0.378[a]	4.91±0.653[a]	<0.001	<0.001
15-HETrE	LOX	0.727±0.479[b]	1.10±0.134[a]	1.58±0.196[a]	<0.001	<0.001
DGLA total		2.59±1.70[b]	4.35±0.505[a]	6.49±0.842[a]	<0.001	<0.001
**AA oxylipins**						
PGE2	COX	0.156±0.0337[b]	0.528±0.197[ab]	1.06±0.310[a]	<0.001	0.010
12-HHTrE	COX	3.18±0.693[b]	7.49±1.89[ab]	13.6±2.68[a]	0.001	0.051
PGB2	COX	9.94±5.48[b]	22.6±2.74[a]	28.9±3.38[a]	<0.001	<0.001
PGF2α	COX	0.865±0.311[b]	2.30±0.360[ab]	2.99±0.315[a]	0.001	0.048
TXB2	COX	0.314±0.0938[b]	0.613±0.132[b]	1.16±0.191[a]	<0.001	<0.001

6k-PGF1α	COX	3.01±0.613^{b}	5.67±1.08^{a}	9.87±1.63^{a}	<0.001	<0.001
AA COX		17.5±6.24^{b}	39.2±5.25^{a}	57.5±5.56^{a}	<0.001	<0.001
14,15-diHETrE	CYP	0.117±0.0558^{ab}	0.165±0.0150^{a}	0.205±0.0197^{a}	<0.001	0.008
5-HETE	LOX	45.6±26.8^{b}	110±14.7^{a}	154±19.8^{a}	<0.001	<0.001
8-HETE	LOX	8.44±3.41^{b}	20.9±2.33^{a}	29.2±3.42^{a}	<0.001	<0.001
11-HETE	LOX	4.95±2.41^{b}	11.9±1.40^{a}	16.4±2.34^{a}	<0.001	<0.001
12-HETE	LOX	3.19±1.22^{b}	7.95±0.900^{a}	11.0±1.21^{a}	<0.001	<0.001
15-HETE	LOX	7.02±3.36^{b}	17.8±1.99^{a}	24.0±2.87^{a}	<0.001	<0.001
15-oxoETE	LOX	2.46±0.660^{b}	5.71±0.770^{a}	6.08±0.602^{a}	<0.001	0.010
8,15-diHETE	LOX	2.16±1.23^{b}	5.37±0.646^{a}	7.50±1.10^{a}	<0.001	<0.001
AA LOX		73.8±38.7^{b}	180±21.5^{a}	248±30.2^{a}	<0.001	<0.001
5-iso PGF2α VI	Non-enz	0.967±0.523^{b}	1.99±0.309^{a}	2.26±0.295	<0.001	0.007
AA total		92.4±45.2^{b}	221±25.5^{a}	308±33.3^{a}	<0.001	<0.001
**ALA oxylipins**						
9-HOTrE	LOX	0.463±0.350^{b}	1.05±0.177^{a}	1.43±0.220^{a}	<0.001	0.007
13-HOTrE	LOX	4.73±3.26^{b}	8.74±1.22^{a}	10.8±1.66^{a}	<0.001	0.003
ALA total		5.19±3.61^{b}	9.79±1.39^{a}	12.2±1.87^{a}	<0.001	<0.001
**EPA oxylipins**						
18-HEPE	CYP	0.319±0.136^{b}	1.42±0.205^{a}	1.74±0.211^{a}	<0.001	<0.001
5-HEPE	LOX	18.9±10.1^{b}	71.2±10.9^{a}	90.7±13.1^{a}	<0.001	<0.001
12-HEPE	LOX	0.288±0.0877^{b}	1.13±0.165^{a}	1.38±0.150^{a}	<0.001	<0.001
15-HEPE	LOX	0.283±0.123^{b}	1.04±0.137^{a}	1.24±0.137^{a}	<0.001	<0.001
EPA LOX		19.5±10.3^{b}	73.3±11.1^{a}	93.3±13.3^{a}	<0.001	<0.001
EPA total		19.8±10.4^{b}	74.8±11.3^{a}	95.0±13.5^{a}	<0.001	<0.001
**DHA oxylipins**						
20-HDoHE	CYP	1.32±0.720^{b}	3.02±0.395^{a}	4.29±0.516^{a}	<0.001	<0.001
					<0.001	<0.001
13-HDoHE	LOX	2.70±1.35^{b}	6.65±0.839^{a}	9.03±1.04^{a}	<0.001	<0.001
14-HDoHE	LOX	0.707±0.360^{b}	1.60±0.197^{a}	2.33±0.239^{a}	<0.001	<0.001
16-HDoHE	LOX	0.876±0.470^{b}	1.92±0.248^{a}	2.79±0.321^{a}	<0.001	<0.001

17-HDoHE	LOX	2.44±1.34[b]	5.48±0.721[a]	7.70±0.830[a]	<0.001	<0.001
DHA LOX		6.72±3.51[b]	15.7±1.98[a]	21.8±2.41[a]	<0.001	<0.001
DHA total		8.04±4.23[b]	18.7±2.37[a]	26.1±2.92[a]	<0.001	<0.001
COX total		17.5±6.24[b]	39.2±5.25[a]	57.5±5.56[a]	<0.001	<0.001
CYP total		1.97±1.01[b]	4.±0.433[a]	4.97±0.610[a]	<0.001	<0.001
LOX total		342±185[b]	710±79.9[a]	987±143[a]	<0.001	<0.001
Total oxylipins		362±192[b]	756±84.0[a]	1053±146[a]	<0.001	-

Values are mean ± standard error. Adjusted *P* values are calculated using the Bonferroni correction for multiple comparisons, with the PUFA totals, Enzyme totals, and PUFA-Enzyme totals calculated separately. AA, arachidonic acid; ALA, α-linolenic acid; COX, cyclooxygenase; CYP, cytochrome P450; DGLA, dihomo-γ-linolenic acid; diHETE, dihydroxy-eicosatetraenoic acid; diHETrE, dihydroxy-eicosatrienoic acid; diHOME, dihydroxy-octadecaenoic acid; DHA, docosahexaenoic acid; GLA, γ-linolenic acid; HDoHE, hydroxy-docosahexaenoic acid; HEPE, hydroxy-eicosapentaenoic acid; HETE, hydroxy-eicosatetraenoic acid; HETrE, hydroxy-eicosatrienoic acid; HHTrE, hydroxyheptadecatrienoic acid; HODE, hydroxy-octadecadienoic acid; HOTrE, hydroxy-octadecatrienoic acid; k, keto; LA, linoleic acid; LOX, lipoxygenase; oxoETE, oxo-eicosatetraenoic acid; oxoODE, oxo-octadecadienoic acid; PG, prostaglandin; triHOME, trihydroxy-octadecenoic acid (9,10,13- triHOME & 9,12,13-triHOME peaks could not be separated); TX, thromboxane.

Table S4.

Aortic Valve Oxylipins by patient ASA use.

Aortic Valve Leaflet Oxylipins (μg/g protein)					
Oxylipin	Enzyme	No ASA<br>n=74	ASA<br>n=25	*P* value	Adjusted *P* value
**LA oxylipins**					
9,10-diHOME	CYP	0.173±0.0227	0.182±0.0369	0.82	>0.99
12,13-diHOME	CYP	0.213±0.0305	0.223±0.0476	0.59	>0.99
LA CYP total		0.386±0.0527	0.405±0.0822	0.54	>0.99
9-HODE	LOX	141±21.2	153±28.8	0.58	>0.99
9-oxoODE	LOX	60.2±8.20	54.6±7.03	0.76	>0.99
13-HODE	LOX	146±22.6	163±35.4	0.53	>0.99
13-oxoODE	LOX	150±27.4	134±19.8	0.38	>0.99
(9,10,13- & 9,12,13-) triHOME	LOX	2.43±0.328	2.48±0.472	0.78	>0.99
LA LOX total		499±75.2	507±88.3	0.57	>0.99
LA total		500±75.3	507±88.3	0.57	>0.99
**GLA oxylipins**					
13-HOTrE-γ	LOX	0.949±0.122	1.04±0.169	0.68	>0.99
**DGLA oxylipins**					
8-HETrE	LOX	3.49±0.464	5.21±0.830	0.083	>0.99
15-HETrE	LOX	1.20±0.149	1.64±0.291	0.14	>0.99
DGLA total		4.69±0.607	6.85±1.11	0.089	0.53
**AA oxylipins**					
PGE2	COX	0.61±0.172	0.731±0.253	0.22	>0.99
12-HHTrE	COX	8.17±1.48	12.4±3.44	0.28	>0.99
PGB2	COX	23.4±2.70	28.1±3.69	0.25	>0.99
PGF2α	COX	2.18±0.229	3.20±0.538	0.13	>0.99
TXB2	COX	0.758±0.119	0.984±0.254	0.55	>0.99
6k-PGF1α	COX	6.81±1.02	8.13±2.04	0.43	>0.99
AA COX		42.0±4.14	53.5±7.86	0.20	>0.99

14,15-diHETrE	CYP	0.169±0.0151	0.219±0.0339	0.13	>0.99
5-HETE	LOX	115±14.2	157±24.9	0.17	>0.99
8-HETE	LOX	22.1±2.52	28.1±3.56	0.18	>0.99
11-HETE	LOX	12.9±1.70	15.2±2.13	0.30	>0.99
12-HETE	LOX	8.25±0.886	10.2±1.30	0.19	>0.99
15-HETE	LOX	18.6±2.15	22.9±2.99	0.22	>0.99
15-oxoETE	LOX	5.47±0.572	5.77±0.659	0.56	>0.99
8,15-diHETE	LOX	5.96±0.804	6.67±0.960	0.33	>0.99
AA LOX		188±22.1	246±35.4	0.18	>0.99
5-iso PGF2α VI	Non-enz	1.97±0.238	2.32±0.429	0.36	>0.99
AA total		232±25.2	302±40.1	0.16	0.96
**ALA oxylipins**					
9-HOTrE	LOX	1.13±0.166	1.36±0.280	0.37	>0.99
13-HOTrE	LOX	9.25±1.23	10.3±2.26	0.68	>0.99
ALA total		10.4±1.39	11.7±2.52	0.65	>0.99
**EPA oxylipins**					
18-HEPE	CYP	1.33±0.152	1.83±0.313	0.21	>0.99
5-HEPE	LOX	67.0±8.16	99.6±20.3	0.19	>0.99
12-HEPE	LOX	1.04±0.105	1.44±0.258	0.21	>0.99
15-HEPE	LOX	0.966±0.100	1.31±0.213	0.21	>0.99
EPA LOX		69.0±8.35	102±20.7	0.19	>0.99
EPA total		70.4±8.48	104±21.0	0.19	>0.99
**DHA oxylipins**					
20-HDoHE	CYP	2.89±0.342	5.11±0.719	0.017	0.62
13-HDoHE	LOX	6.51±0.73	9.94±1.43	0.061	>0.99
14-HDoHE	LOX	1.60±0.169	2.57±0.346	0.025	0.88
16-HDoHE	LOX	1.94±0.228	3.09±0.430	0.036	>0.99
17-HDoHE	LOX	5.42±0.598	8.59±1.23	0.048	>0.99
DHA LOX		15.5±1.71	24.2±3.42	0.048	0.29

DHA total	18.4±2.05	29.3±4.13	0.040	0.24
COX total	42.0±4.14	53.5±7.86	0.20	0.60
CYP total	4.77±0.529	7.57±1.07	0.040	0.12
LOX total	788±102	899±139	0.39	>0.99
Total oxylipins	837±106	962±145	0.36	-

Values are mean ± standard error. Adjusted *P* values are calculated using the Bonferroni correction for multiple comparisons, with the PUFA totals, Enzyme totals, and PUFA-Enzyme totals calculated separately. AA, arachidonic acid; ALA, α-linolenic acid; COX, cyclooxygenase; CYP, cytochrome P450; DGLA, dihomo-γ-linolenic acid; diHETE, dihydroxy-eicosatetraenoic acid; diHETrE, dihydroxy-eicosatrienoic acid; diHOME, dihydroxy-octadecaenoic acid; DHA, docosahexaenoic acid; GLA, γ-linolenic acid; HDoHE, hydroxy-docosahexaenoic acid; HEPE, hydroxy-eicosapentaenoic acid; HETE, hydroxy-eicosatetraenoic acid; HETrE, hydroxy-eicosatrienoic acid; HHTrE, hydroxyheptadecatrienoic acid; HODE, hydroxy-octadecadienoic acid; HOTrE, hydroxy-octadecatrienoic acid; k, keto; LA, linoleic acid; LOX, lipoxygenase; oxoETE, oxo-eicosatetraenoic acid; oxoODE, oxo-octadecadienoic acid; PG, prostaglandin; triHOME, trihydroxy-octadecenoic acid (9,10,13- triHOME & 9,12,13-triHOME peaks could not be separated); TX, thromboxane.

Table S5.

Plasma oxylipins by aortic valve stenosis severity determined by patient transvalvular mean pressure gradient.

		Plasma Oxylipins (ng/ml)					
Oxylipin	Enzyme	Control n=44	Mild n=5	Moderate n=27	Severe n=31	*P* value	Adjusted *P* value
**LA oxylipins**							
9-HODE	LOX	7.75±0.659^{b}	16.7±3.37^{a}	17.4±1.44^{a}	20.7±1.62^{a}	<0.001	<0.001
9-oxoODE	LOX	0.993±0.101^{b}	4.62±0.663^{a}	7.63±1.44^{a}	6.77±0.681^{a}	<0.001	<0.001
13-HODE	LOX	3.43±0.256^{b}	7.97±1.65^{a}	7.61±0.545^{a}	9.38±0.635^{a}	<0.001	<0.001
13-oxoODE	LOX	0.444±0.0649^{b}	5.15±0.559^{a}	7.70±1.28^{a}	9.54±1.46^{a}	<0.001	<0.001
(9,10,13- & 9,12,13-) triHOME	LOX	4.39±0.396^{a}	0.175±0.0349^{b}	0.216±0.0185^{b}	0.316±0.0353^{b}	<0.001	<0.001
LA total		17.0±1.20^{b}	34.6±5.99^{a}	40.6±4.05^{a}	46.7±4.09^{a}	<0.001	<0.001
**GLA oxylipins**							
13-HOTrE-γ	LOX	9.45±0.705^{a}	0.0979±0.0267^{b}	0.156±0.0212^{b}	0.152±0.0137^{b}	<0.001	<0.001
**DGLA oxylipins**							
8-HETrE	LOX	0.347±0.0431^{a}	0.120±0.0385^{b}	0.144±0.0194^{ab}	0.135±0.0106^{ab}	<0.001	<0.001
15-HETrE	LOX	0.190±0.0203	0.0886±0.0194	0.149±0.0106	0.141±0.0103	0.24	>0.99
DGLA total		0.537±0.0546^{a}	0.208±0.0570^{b}	0.292±0.0280^{ab}	0.276±0.0196^{ab}	<0.001	0.002
**AA oxylipins**							
5-HETE	LOX	0.917±0.233^{b}	0.984±0.266^{ab}	1.17±0.111^{a}	1.25±0.0849	<0.001	<0.001
8-HETE	LOX	23.2±1.46^{a}	0.381±0.115^{b}	0.537±0.0700^{b}	0.482±0.0347^{b}	<0.001	<0.001
11-HETE	LOX	0.264±0.0356	0.126±0.0279	0.165±0.0142	0.161±0.0151	0.14	3.98
12-HETE	LOX	0.255±0.0492^{b}	4.18±2.25^{a}	6.33±1.06^{a}	7.41±1.30^{a}	<0.001	<0.001
tetranor 12-HETE	LOX	0.659±0.0637^{b}	0.252±0.0921^{a}	0.303±0.0575^{a}	0.270±0.0317^{a}	<0.001	<0.001
15-HETE	LOX	0.472±0.0539^{b}	0.560±0.157^{ab}	0.817±0.108^{ab}	1.04±0.204^{a}	<0.001	<0.001
AA total		25.7±1.67^{a}	6.48±2.70^{b}	9.32±1.13^{b}	10.6±1.38^{b}	<0.001	<0.001
**ALA oxylipins**							
9-HOTrE	LOX	0.393±0.0288^{b}	2.82±0.782^{a}	2.99±0.274^{a}	3.70±0.316^{a}	<0.001	<0.001
9-oxoOTrE	LOX	1.73±0.184^{a}	0.613±0.106^{b}	1.10±0.168^{ab}	1.16±0.153^{ab}	0.005	0.14

13-HOTrE	LOX	0.302±0.0434^{b}	1.21±0.341^{a}	1.38±0.121^{a}	1.71±0.143^{a}	<0.001	<0.001
ALA total		2.43±0.233^{b}	4.64±1.21^{a}	5.48±0.480^{a}	6.58±0.567^{a}	<0.001	<0.001
**EPA oxylipins**							
5-HEPE	LOX	0.733±0.0781^{a}	0.287±0.0769^{b}	0.448±0.0986^{ab}	0.489±0.0516^{ab}	0.010	0.28
12-HEPE	LOX	0.727±0.0624^{a}	0.915±0.396^{a}	1.85±0.305^{a}	3.23±0.660^{a}	0.012	0.34
15-HEPE	LOX	0.176±0.0156	0.0824±0.0164	0.149±0.0119	0.177±0.0229	0.31	>0.99
EPA total		1.64±0.124^{ab}	1.28±0.459^{b}	2.45±0.322^{ab}	3.89±0.705^{a}	0.042	0.25
**DPA oxylipins**							
17k-DPA	LOX	0.435±0.0653^{b}	6.78±1.97^{a}	13.2±1.46^{a}	12.4±1.26^{a}	<0.001	<0.001
**DHA oxylipins**							
4-HDoHE	LOX	0.229±0.0239^{b}	0.574±0.131^{a}	0.770±0.0639^{a}	0.958±0.112^{a}	<0.001	<0.001
7-HDoHE	LOX	0.313±0.0600^{b}	0.263±0.0499^{ab}	0.377±0.0414^{a}	0.377±0.0364^{a}	0.004	0.11
8-HDoHE	LOX	0.384±0.0791^{b}	0.368±0.104^{ab}	0.480±0.0411^{a}	0.567±0.0561^{a}	<0.001	<0.001
10-HDoHE	LOX	1.15±0.182^{a}	0.165±0.0537^{b}	0.269±0.0310^{b}	0.316±0.0401^{b}	<0.001	<0.001
11-HDoHE	LOX	5.23±0.645^{a}	0.230±0.0752^{c}	0.353±0.0391^{bc}	0.455±0.0590^{b}	<0.001	<0.001
14-HDoHE	LOX	1.65±0.277^{a}	1.92±0.825^{a}	4.02±0.694^{a}	5.34±0.912^{a}	0.007	0.20
16-HDoHE	LOX	0.549±0.0842^{a}	0.0994±0.0425^{b}	0.207±0.0348^{ab}	0.269±0.0444^{ab}	<0.001	0.001
17-HDoHE	LOX	1.81±0.123^{a}	0.377±0.113^{c}	0.619±0.0572^{bc}	0.715±0.0745^{b}	<0.001	<0.001
DHA total		11.3±1.05^{a}	4.00±1.28^{b}	7.09±0.845^{ab}	8.99±1.22^{ab}	0.002	0.011
Total oxylipins		68.5±4.22^{ab}	58.1±11.7^{b}	78.6±5.46^{ab}	89.6±7.04^{a}	0.019	-

Values are mean ± standard error. Adjusted *P* values are calculated using the Bonferroni correction for multiple comparisons, with the PUFA totals calculated separately. AA, arachidonic acid; ALA, α-linolenic acid; DGLA, dihomo-γ-linolenic acid; DHA, docosahexaenoic acid; DPA, docosapentaenoic acid; GLA, γ-linolenic acid; HDoHE, hydroxy-docosahexaenoic acid; HEPE, hydroxy-eicosapentaenoic acid; HETE, hydroxy-eicosatetraenoic acid; HETrE, hydroxy-eicosatrienoic acid; HODE, hydroxy-octadecadienoic acid; HOTrE, hydroxy-octadecatrienoic acid; LA, linoleic acid; LOX, lipoxygenase; oxoODE, oxo-octadecadienoic acid; oxoOTrE, oxo-octadecatrienoic acid; triHOME, trihydroxy-octadecenoic acid (9,10,13- triHOME & 9,12,13-triHOME peaks could not be separated).

Table S6.

Aortic valve leaflet oxylipins grouped by patient aortic valve stenosis severity (transvalvular mean pressure gradient) and sex.

Aortic Valve Leaflet Oxylipins Sex x MPG-Severity (µg/g protein)							
Oxylipin	ENZ	Female-Mild	Female-Moderate	Female-severe	Male-mild	Male-moderate	Male-severe
		n=3	n=16	n=12	n=12	n=23	n=33
**LA oxylipins**							
9,10-diHOME	CYP	0.0294±0.0120	0.214±0.0574	0.226±0.0732	0.127±0.0567	0.116±0.0182	0.187±0.0303
12,13-diHOME	CYP	0.0295±0.0274	0.262±0.0770	0.279±0.0954	0.141±0.0714	0.169±0.0298	0.215±0.0404
LA CYP total	CYP	0.0589±0.0394	0.476±0.134	0.505±0.166	0.268±0.128	0.285±0.0442	0.402±0.0702
9-HODE	LOX	7.50±1.57	134±26.3	267±88.7	85.9±53.1	112±17.6	140±23.5
9-oxoODE	LOX	8.62±4.47	69.9±15.8	87.5±19.4	31.2±9.36	45.6±6.41	58.6±12.8
13-HODE	LOX	5.12±1.05	139±27.5	281±93.1	95.9±65.8	110±17.4	147±27.2
13-oxoODE	LOX	12.3±6.71	149±35.4	253±97.2	69.2±29.9	108±16.7	150±39.3
(9,10,13- & 9,12,13-) triHOME	LOX	0.182±0.0589	2.87±0.658	3.53±1.16	1.48±0.808	1.95±0.299	2.39±0.419
LA LOX total	LOX	33.7±13.4	496±99.8	892±296	284±158	377±55.3	499±93.2
LA total		33.8±13.4	496±99.9	893±297	284±158	377±55.3	499±93.3
**GLA oxylipins**							
13-HOTrE-γ	LOX	0.0421±0.000588	0.952±0.177	1.66±0.347	0.370±0.187	0.924±0.156	0.930±0.183
**DGLA oxylipins**							
8-HETrE	LOX	0.176±0.0219	3.35±0.571	7.19±1.73	2.29±1.51	3.18±0.514	4.08±0.587
15-HETrE	LOX	0.0729±0.0287	1.21±0.222	2.27±0.502	0.891±0.594	1.02±0.170	1.33±0.182
DGLA total		0.249±0.0454	4.56±0.773	9.47±2.21	3.18±2.11	4.2±0.679	5.41±0.763
**AA oxylipins**							
PGE2	COX	0.0728±0.0275	0.296±0.095	0.425±0.0714	0.177±0.0397	0.690±0.326	1.29±0.416
12-HHTrE	COX	2.15±1.27	5.97±1.96	6.34±0.957	3.43±0.813	8.55±2.93	16.2±3.55
PGB2	COX	0.365±0.218	21.4±3.82	40.3±8.06	12.3±6.72	23.5±3.87	24.7±3.36

PGF2α	COX	0.363±0.147	1.91±0.454	2.61±0.378	0.99±0.381	2.57±0.522	3.13±0.407
TXB2	COX	0.170±0.119	0.465±0.125	0.674±0.0870	0.35±0.113	0.716±0.207	1.34±0.253
6k-PGF1α	COX	2.49±1.71	5.00±1.40	5.36±1.05	3.14±0.676	6.14±1.56	11.5±2.13
AA COX		5.61±3.26	35.1±6.75	55.7±7.66	20.4±7.58	42.1±7.62	58.2±7.11
14,15-diHETrE	CYP	0.0369±0.000429	0.161±0.0222	0.257±0.047	0.137±0.0691	0.167±0.0206	0.186±0.0202
5-HETE	LOX	3.27±0.749	86.4±13.0	219±43.1	56.2±33.1	127±22.8	130±20.9
8-HETE	LOX	1.55±0.332	20.2±3.08	40.2±8.28	10.2±4.14	21.3±3.37	25.2±3.39
11-HETE	LOX	0.929±0.159	11.6±2.02	22.9±4.93	5.96±2.96	12.2±1.95	14.1±2.56
12-HETE	LOX	0.636±0.0798	7.53±1.21	14.3±2.69	3.83±1.47	8.24±1.29	9.85±1.29
15-HETE	LOX	1.09±0.0869	17.1±2.74	34.1±7.01	8.50±4.12	18.4±2.84	20.3±2.78
15-oxoETE	LOX	1.36±0.565	6.71±1.54	7.09±1.00	2.73±0.803	5.01±0.746	5.71±0.733
8,15-diHETE	LOX	0.176±0.0755	5.12±0.968	10.7±2.77	2.65±1.51	5.54±0.880	6.33±1.07
AA LOX		9.01±1.32	155±22.5	349±66.4	90.1±47.6	197±32.9	212±31.7
5-iso PGF2α VI	Non-ENZ	0.0873±0.0201	1.73±0.331	3.04±0.570	1.19±0.642	2.17±0.473	1.98±0.336
AA total		14.7±4.57	192±28.1	408±73.4	112±55.4	242±38.6	272±35.4
**ALA oxylipins**							
9-HOTrE	LOX	<LOQ	1.32±0.301	2.12±0.512	0.578±0.435	0.861±0.211	1.18±0.225
13-HOTrE	LOX	0.249±0.0464	10.7±2.15	14.9±3.80	5.85±4.04	7.37±1.41	9.28±1.76
ALA total		0.249±0.0464	12.0±2.43	17.0±4.29	6.42±4.47	8.23±1.61	10.5±1.98
**EPA oxylipins**							
18-HEPE	CYP	0.0808±0.00566	1.44±0.252	2.55±0.550	0.378±0.167	1.40±0.305	1.44±0.188
5-HEPE	LOX	1.81±0.389	60.1±11.2	140±37.8	23.2±12.4	78.9±16.7	72.8±10.2
12-HEPE	LOX	0.111±0.00177	1.09±0.193	1.83±0.392	0.333±0.107	1.15±0.249	1.22±0.142
15-HEPE	LOX	0.0591±0.0128	1.08±0.168	1.73±0.351	0.339±0.150	1.01±0.205	1.06±0.127
EPA LOX		1.98±0.375	62.3±11.5	143±38.5	23.8±12.6	81.0±17.1	75.1±10.4
EPA total		2.06±0.380	63.7±11.7	146±39.0	24.2±12.8	82.4±17.4	76.6±10.6
**DHA oxylipins**							
20-HDoHE	CYP	0.193±0.0242	2.62±0.421	6.27±1.48	1.60±0.889	3.30±0.603	3.57±0.408

13-HDoHE	LOX	0.488±0.0765	5.98±0.856	13.1±3.01	3.25±1.66	7.12±1.30	7.56±0.804
14-HDoHE	LOX	0.121±0.0247	1.46±0.215	3.20±0.646	0.854±0.443	1.71±0.301	2.01±0.207
16-HDoHE	LOX	0.116±0.0189	1.69±0.279	4.04±0.898	1.07±0.579	2.08±0.375	2.33±0.261
17-HDoHE	LOX	0.365±0.0371	4.90±0.844	10.9±2.38	2.95±1.65	5.87±1.08	6.55±0.655
DHA LOX	LOX	1.09±0.0688	14.0±2.15	31.2±6.91	8.13±4.33	16.8±3.03	18.5±1.90
DHA total		1.28±0.0501	16.7±2.55	37.4±8.38	9.73±5.22	20.1±3.62	22.0±2.29
COX total		5.61±3.26	35.1±6.75	55.7±7.66	20.4±7.58	42.1±7.62	58.2±7.11
CYP total		0.370±0.0453	4.69±0.696	9.59±2.17	2.38±1.24	5.16±0.926	5.6±0.635
LOX total		46.3±14.8	744±130	1443±377	416±228	685±103	821±131
Total oxylipins		52.4±18.1	786±136	1512±387	440±237	735±109	886±135

Values are mean ± standard error. AA, arachidonic acid; ALA, α-linolenic acid; COX, cyclooxygenase; CYP, cytochrome P450; DGLA, dihomo-γ-linolenic acid; diHETE, dihydroxy-eicosatetraenoic acid; diHETrE, dihydroxy-eicosatrienoic acid; diHOME, dihydroxy-octadecaenoic acid; DHA, docosahexaenoic acid; ENZ, Enzyme; GLA, γ-linolenic acid; HDoHE, hydroxy-docosahexaenoic acid; HEPE, hydroxy-eicosapentaenoic acid; HETE, hydroxy-eicosatetraenoic acid; HETrE, hydroxy-eicosatrienoic acid; HHTrE, hydroxyheptadecatrienoic acid; HODE, hydroxy-octadecadienoic acid; HOTrE, hydroxy-octadecatrienoic acid; k, keto; LA, linoleic acid; LOX, lipoxygenase; oxoETE, oxo-eicosatetraenoic acid; oxoODE, oxo-octadecadienoic acid; PG, prostaglandin; triHOME, trihydroxy-octadecenoic acid (9,10,13- triHOME & 9,12,13-triHOME peaks could not be separated); TX, thromboxane.

Table S7.

Plasma oxylipins grouped by patient aortic valve stenosis severity (transvalvular mean pressure gradient) and sex.

		Plasma Oxylipins (ng/ml)							
Oxylipin	ENZ	Female-Control n=13	Female-Mild n=1	Female-Moderate n=11	Female-Severe n=22	Male-Control n=18	Male-Mild n=4	Male-Moderate n=16	Male-Severe n=19
**LA oxylipins**									
9-HODE	LOX	5.53±0.418	23.4	17.4±1.92	20.4±2.13	7.59±0.986	15.0±3.77	17.5±2.10	21±2.53
9-oxoODE	LOX	0.712±0.124	5.20	9.6±3.25	6.97±1.05	0.947±0.153	4.47±0.835	6.28±0.955	6.54±0.849
13-HODE	LOX	3.14±0.254	11.4	7.66±0.738	9.28±0.830	3.41±0.368	7.13±1.83	7.58±0.787	9.49±1.00
13-oxoODE	LOX	0.478±0.0984	5.99	8.93±2.91	9.96±2.30	0.481±0.108	4.94±0.669	6.86±0.900	9.07±1.76
(9,10,13- & 9,12,13-) triHOME	LOX	3.67±0.417	0.200	0.227±0.0334	0.295±0.0481	3.99±0.491	0.168±0.0443	0.208±0.0218	0.340±0.0528
LA total		13.5±0.971	46.2	43.8±8.12	46.9±6.05	16.4±1.67	31.7±6.78	38.4±4.11	46.5±5.54
**GLA oxylipins**									
13-HOTrE-γ	LOX	9.61±1.05	0.0768	0.171±0.0394	0.158±0.0205	8.77±1.02	0.103±0.0338	0.145±0.0241	0.145±0.0180
**DGLA oxylipins**									
8-HETrE	LOX	0.352±0.0693	0.106	0.165±0.0462	0.142±0.0131	0.307±0.0659	0.123±0.0495	0.129±0.00942	0.127±0.0172
15-HETrE	LOX	0.160±0.0241	0.0604	0.147±0.0195	0.146±0.0122	0.198±0.0337	0.0957±0.0233	0.150±0.0123	0.134±0.0174
DGLA total		0.512±0.0762	0.167	0.312±0.0644	0.288±0.0245	0.504±0.0872	0.219±0.0724	0.279±0.0193	0.261±0.0319
**AA oxylipins**									
5-HETE	LOX	0.870±0.482	1.16	1.15±0.182	1.28±0.104	0.739±0.224	0.939±0.338	1.19±0.143	1.22±0.141
8-HETE	LOX	19.6±2.05	0.255	0.604±0.164	0.512±0.0491	23.8±2.43	0.413±0.143	0.491±0.0417	0.448±0.0488
11-HETE	LOX	0.193±0.0387	0.0974	0.166±0.0232	0.161±0.0155	0.222±0.0468	0.134±0.0348	0.165±0.0185	0.162±0.0279
12-HETE	LOX	0.234±0.0749	1.14	6.39±1.63	8.32±2.10	0.204±0.0546	4.94±2.74	6.29±1.43	6.35±1.42
tetranor 12-HETE	LOX	0.496±0.0897	0.393	0.43±0.121	0.281±0.0489	0.650±0.0728	0.217±0.110	0.215±0.0408	0.257±0.0397
15-HETE	LOX	0.473±0.115	0.401	0.871±0.200	0.785±0.0792	0.463±0.0878	0.600±0.196	0.780±0.125	1.33±0.428
AA total		21.9±2.55	3.45	9.6±1.64	11.3±2.18	26.1±2.63	7.24±3.34	9.13±1.58	9.77±1.63

**ALA oxylipins**									
9-HOTrE	LOX	0.364±0.0460	5.38	3.16±0.348	3.75±0.485	0.393±0.0512	2.18±0.581	2.88±0.403	3.65±0.400
9-oxoOTrE	LOX	1.63±0.217	0.970	1.26±0.357	1.25±0.259	1.76±0.388	0.523±0.0731	0.996±0.149	1.06±0.141
13-HOTrE	LOX	0.250±0.0473	2.23	1.39±0.138	1.67±0.215	0.273±0.0574	0.956±0.293	1.38±0.184	1.77±0.187
ALA total		2.24±0.282	8.58	5.8±0.725	6.66±0.905	2.43±0.464	3.66±0.901	5.26±0.653	6.48±0.659
**EPA oxylipins**									
5-HEPE	LOX	0.564±0.0935	0.518	0.610±0.223	0.540±0.0790	0.677±0.107	0.23±0.0658	0.337±0.0599	0.431±0.0631
12-HEPE	LOX	0.650±0.124	0.936	2.1±0.551	3.59±1.01	0.694±0.0895	0.910±0.511	1.68±0.358	2.81±0.827
15-HEPE	LOX	0.150±0.0283	0.136	0.144±0.0211	0.171±0.0248	0.173±0.0241	0.069±0.0121	0.152±0.0144	0.184±0.0409
EPA total		1.36±0.196	1.59	2.85±0.575	4.30±1.06	1.54±0.164	1.21±0.584	2.17±0.372	3.42±0.912
**DPA oxylipins**									
17k-DPA	LOX	0.480±0.0936	13.4	12.4±2.26	14.6±1.78	0.338±0.0778	5.12±1.38	13.8±1.95	9.93±1.64
**DHA oxylipins**									
4-HDoHE	LOX	0.210±0.0276	0.911	0.735±0.134	0.931±0.153	0.258±0.0386	0.490±0.129	0.794±0.0601	0.990±0.170
7-HDoHE	LOX	0.302±0.0767	0.409	0.364±0.082	0.400±0.0531	0.228±0.0569	0.227±0.0438	0.386±0.0440	0.350±0.0497
8-HDoHE	LOX	0.602±0.262	0.628	0.465±0.0793	0.572±0.0869	0.284±0.0150	0.303±0.104	0.49±0.0453	0.561±0.0699
10-HDoHE	LOX	0.894±0.166	0.273	0.259±0.0483	0.324±0.0515	1.02±0.229	0.138±0.0598	0.276±0.0416	0.307±0.0643
11-HDoHE	LOX	3.81±1.08	0.416	0.369±0.0726	0.482±0.0879	5.02±0.744	0.184±0.0762	0.341±0.0452	0.423±0.0785
14-HDoHE	LOX	1.64±0.339	1.99	3.98±1.04	5.23±1.22	1.44±0.254	1.90±1.06	4.04±0.958	5.45±1.40
16-HDoHE	LOX	0.426±0.0687	0.0995	0.204±0.0518	0.263±0.0609	0.457±0.103	0.0994±0.0549	0.209±0.0481	0.275±0.0666
17-HDoHE	LOX	1.58±0.149	0.741	0.556±0.0824	0.725±0.0974	1.81±0.206	0.286±0.0871	0.662±0.0784	0.703±0.117
DHA total		9.46±1.33	5.47	6.93±1.23	8.93±1.58	10.5±0.905	3.63±1.58	7.20±1.18	9.06±1.93
Total oxylipins		59.1±5.59	78.9	81.9±9.99	93.1±9.54	66.6±5.87	52.9±13.6	76.3±6.37	85.5±10.6

Values are mean ± standard error. AA, arachidonic acid; ALA, α-linolenic acid; DGLA, dihomo-γ-linolenic acid; DHA, docosahexaenoic acid; DPA, docosapentaenoic acid; ENZ, Enzyme; GLA, γ-linolenic acid; HDoHE, hydroxy-docosahexaenoic acid; HEPE, hydroxy-eicosapentaenoic acid; HETE, hydroxy-eicosatetraenoic acid; HETrE, hydroxy-eicosatrienoic acid; HODE, hydroxy-octadecadienoic acid; HOTrE, hydroxy-octadecatrienoic acid; LA, linoleic acid; LOX, lipoxygenase; oxoODE, oxo-octadecadienoic acid; oxoOTrE, oxo-octadecatrienoic acid; triHOME, trihydroxy-octadecenoic acid (9,10,13- triHOME & 9,12,13-triHOME peaks could not be separated).

Figure S1.
Example chromatograms of oxylipin peak selection.

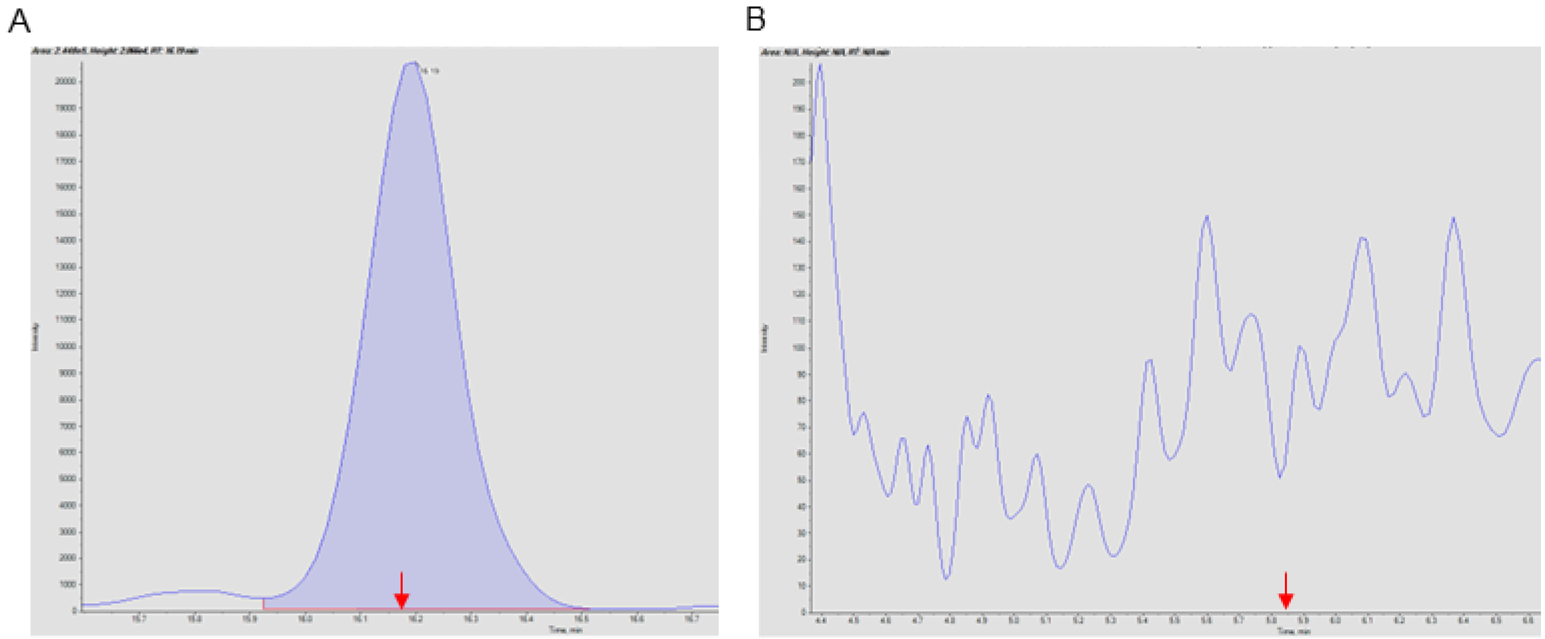

A) Chromatogram of 15-hydroxy-eicosatetraenoic acid. Peak selected at **e**xpected retention time of 16.17 min exceeds 5-times baseline. **B) Chromatogram of resolvin E1.** Peak not selected at expected retention time of 5.84 min, does not exceed baseline.

Major Resources Table

In order to allow validation and replication of experiments, all essential research materials listed in the Methods should be included in the Major Resources Table below. Authors are encouraged to use public repositories for protocols, data, code, and other materials and provide persistent identifiers and/or links to repositories when available. Authors may add or delete rows as needed.

Animals (in vivo studies)

Species	Vendor or Source	Background Strain	Sex	Persistent ID / URL
NA				

Genetically Modified Animals

	Species	Vendor or Source	Background Strain	Other Information	Persistent ID / URL
**Parent - Male**	NA				
**Parent - Female**					

Antibodies

Target antigen	Vendor or Source	Catalog #	Working concentration	Lot # (preferred but not required)	Persistent ID / URL
NA					

DNA/cDNA Clones

Clone Name	Sequence	Source / Repository	Persistent ID / URL
NA			

Cultured Cells

Name	Vendor or Source	Sex (F, M, or unknown)	Persistent ID / URL
NA			

Data & Code Availability

Description	Source / Repository	Persistent ID / URL
NA		

Other

Description	Source / Repository	Persistent ID / URL
NA		

DOI [to be added]

ARRIVE GUIDELINES

The ARRIVE guidelines (https://arriveguidelines.org/) are a checklist of recommendations to improve the reporting of research involving animals. Key elements of the study design should be included below to better enable readers to scrutinize the research adequately, evaluate its methodological rigor, and reproduce the methods or findings.

Study Design

Groups	Sex	Age	Number (prior to experiment)	Number (after termination)	Littermates (Yes/No)	Other description
Group 1 (Control)	NA					
Group 2	NA					
Add more if needed	NA					

Sample Size: Please explain how the sample size was decided Please provide details of any a *prior* sample size calculation, if done.

Inclusion Criteria

NA

Exclusion Criteria

NA

Randomization

NA

Blinding

NA

DOI [to be added]
